# A Major Locus Controls a Genital Shape Difference Involved in Reproductive Isolation Between *Drosophila yakuba* and *Drosophila santomea*

**DOI:** 10.1534/g3.115.023481

**Published:** 2015-10-27

**Authors:** Alexandre E. Peluffo, Isabelle Nuez, Vincent Debat, Rosina Savisaar, David L. Stern, Virginie Orgogozo

**Affiliations:** * UMR7592, Institut Jacques Monod, CNRS, Univ Paris Diderot, Sorbonne Paris Cité, 75013 Paris, France; † UMR MNHN/MNHN/UPMC/EPHE 7205, Institut de Systématique, Evolution et Biodiversité, Muséum National d’Histoire Naturelle, 75005 Paris, France; ‡ Janelia Research Campus, Howard Hughes Medical Institute, Ashburn, Virginia 20147

**Keywords:** genitalia, QTL, *Drosophila*, shape, lock-and-key

## Abstract

Rapid evolution of genitalia shape, a widespread phenomenon in animals with internal fertilization, offers the opportunity to dissect the genetic architecture of morphological evolution linked to sexual selection and speciation. Most quantitative trait loci (QTL) mapping studies of genitalia divergence have focused on *Drosophila melanogaster* and its three most closely related species, *D. simulans*, *D. mauritiana*, and *D. sechellia*, and have suggested that the genetic basis of genitalia evolution involves many loci. We report the first genetic study of male genitalia evolution between *D. yakuba* and *D. santomea*, two species of the *D. melanogaster* species subgroup. We focus on male ventral branches, which harm females during interspecific copulation. Using landmark-based geometric morphometrics, we characterized shape variation in parental species, F1 hybrids, and backcross progeny and show that the main axis of shape variation within the backcross population matches the interspecific variation between parental species. For genotyping, we developed a new molecular method to perform multiplexed shotgun genotyping (MSG), which allowed us to prepare genomic DNA libraries from 365 backcross individuals in a few days using little DNA. We detected only three QTL, one of which spans 2.7 Mb and exhibits a highly significant effect on shape variation that can be linked to the harmfulness of the ventral branches. We conclude that the genetic architecture of genitalia morphology divergence may not always be as complex as suggested by previous studies.

Male genitalia evolve faster than other organs in animals with internal fertilization (Eberhard 1985, 2010. Most explanations of this rapid evolution have involved sexual selection (Arnqvist 1998; Hosken and Stockley 2004). According to the cryptic female choice (CFC) hypothesis, male genitalia are thought to evolve to increase the male’s chances of being chosen by females. The sexual antagonist coevolution (SAC) hypothesis predicts that male genitalia evolve to prevent females from mating with other males. Other hypotheses not involving sexual selection have been proposed, such as the “lock-and-key” hypothesis (Dufour 1844; Masly 2012), where male genitalia evolve toward high match with female genitalia of their own species and high divergence with female genitalia of other species. Diversified genitalia may play an essential role in speciation, whether acting at the onset of the lineage splitting process or after speciation to reinforce gene flow barriers between species (Coyne and Orr 2004; Eberhard 2010; Masly 2012).

Genitalia provide a unique opportunity to understand the evolution of body shape in relation to selection and speciation, a key challenge for modern biology (Frankino *et al.* 2009; Klingenberg 2010). To better understand how genitalia diversify, it is important to characterize their genetic architecture. Surprisingly, few studies have examined the genetic basis of animal genitalia evolution. Crosses in lab-controlled environments have revealed significant levels of heritability for genital traits of water striders (Preziosi and Roff 1998), dung beetles (Simmons and Garcia-Gonzalez 2011), and rainbow fish (Evans *et al.* 2013). Using genetic markers, quantitative trait loci (QTL) responsible for genitalia evolution have been identified in only two taxa to our knowledge, carabid beetles and *Drosophila* flies. One of the first quantitative trait loci mapping studies focused on the difference in the male posterior lobe shape between the closely related species *Drosophila simulans* and *Drosophila mauritiana* (Liu *et al.* 1996). Using Fourier contour analysis and 18 genetic markers, the authors found 9 loci, with each spanning approximately 20 cM, whose effects were additive. With larger sample sizes and twice as many markers, Zeng *et al.* (2000) confirmed that the genetic architecture of posterior lobe shape was polygenic, involving at least 19 loci, and that the effect of each locus was mainly additive, with low epistasis. All but one locus were associated with phenotypic changes in the same direction (*D. simulans* alleles associated with lobe size increase), suggesting that this interspecific difference in shape did not evolve through genetic drift, but rather through directional selection (Orr 1998). Following in the footsteps of these landmarks papers, other genitalia QTL mapping analyses examined different genitalia traits: anal plate size and clasper size between *D. simulans* and *D. mauritiana* (True *et al.* 1997), multiple genitalia traits between *D. simulans* and *Drosophila sechellia* (Macdonald and Goldstein 1999),genitalia lobe shape variation within *D. melanogaster* populations (McNeil *et al.* 2011; Takahara and Takahashi 2015), and distiphallus shape and size in distinct lines of *Drosophila montana* (Schäfer *et al.* 2011). Recently, in a QTL mapping study between *D. mauritiana* and *D. simulans*, Tanaka *et al.* (2015) found three loci for male clasper bristle number and three loci for male clasper size; epistatic interactions were found for male clasper bristle number, but not for male clasper size. Altogether, these studies found that the genetic architecture of genitalia shape evolution involves multiple loci displaying little epistasis (Orr 2001).

In contrast, a recent QTL analysis between two closely related species of carabid beetles reported that only a few loci determine the difference in length, width, and weight of the male copulatory piece (Sasabe *et al.* 2010). Because all the other QTL studies of animal genitalia have focused on the four species of the *D. melanogaster* complex (*D. melanogaster*, *D. simulans*, *D. sechellia*, and *D. mauritiana*), besides the study of Schäfer *et al.* (2011) on *D. montana*, the result that genitalia architecture is controlled by many additive loci may not be a general rule. Moreover, the studies performed on the *D. melanogaster* complex have mainly focused on posterior lobe shape even though its link with reproductive isolation and adaptive evolution continues to be debated. A recent study has suggested that although the lobe itself is important for mating, its shape variation within and across species does not appear to be linked to reproductive isolation or sexual selection (LeVasseur-Viens *et al.* 2015).

Here, we perform the first QTL mapping of genitalia divergence between *Drosophila yakuba* and *D. santomea*, and we focus on a trait that has been linked to reproductive success, the shape of the ventral branches. The *yakuba* complex (*Drosophila teissieri*, *D. yakuba*, and *D. santomea*) is closely related to the well-studied *melanogaster* complex (David *et al.* 2007). *D. yakuba* and *D. santomea* can be crossed to generate fertile F1 females, and several studies have performed genetic analysis of phenotypic differences between these species (Coyne *et al.* 2004; Rebeiz *et al.* 2009; Cande *et al.* 2012). *D. yakuba* is found on the African continent, whereas *D. santomea* is endemic to the island of Saõ Tomé (Lachaise *et al.* 2000; David *et al.* 2007). Their evolutionary divergence is estimated to have occurred 0.5 million years ago through the migration of ancestral *D. yakuba* to the island of Saõ Tomé (Llopart *et al.* 2005a). Current Saõ Tomé populations of *D. yakuba* are thought to have been reintroduced very recently on the island and are found in the low, drier, and inhabited lands, whereas *D. santomea* lives in the misty forest of the highlands (Lachaise *et al.* 2000). Both species can be found at mid altitudes and hybrids have been collected (Lachaise *et al.* 2000; Llopart *et al.* 2005a,b). Multiple reproductive isolating mechanisms have been identified between the two species, such as genetic incompatibilities (Coyne *et al.* 2004; Moehring *et al.* 2006), ecological niche divergence (Matute *et al.* 2009), and behavioral and physiological differences (Matute 2010; Cande *et al.* 2012).

One of the reproductive isolating mechanisms between *D. yakuba* and *D. santomea* involves a difference in ventral branches shape in the male genitalia and is the most conspicuous difference in genitalia morphology between males of the two species (Lachaise *et al.* 2000). In *D. yakuba*, spiny ventral branches located above the aedaegus (*i.e.*, the insect phallus, see Figure 1, A and C) insert inside female protective pouches during mating. In *D. santomea*, the male spines (Figure 1B) and female pouches are absent (Kamimura and Mitsumoto 2012a; Kamimura 2012; Yassin and Orgogozo 2013). These structures play important roles during mating. For example, Kamimura and Mitsumoto (2012a) observed that *D. santomea* females mated with *D. yakuba* males possessed significantly more wounded pouches than unmated females or females that had mated with conspecific *D. santomea* males. Moreover, the wounds were found bilaterally, strongly suggesting that the damage was caused by the pair of spines on *D. yakuba* male genitalia. It is not yet known if the spines and pouches play a role in intraspecific sexual selection.

**Figure 1:**
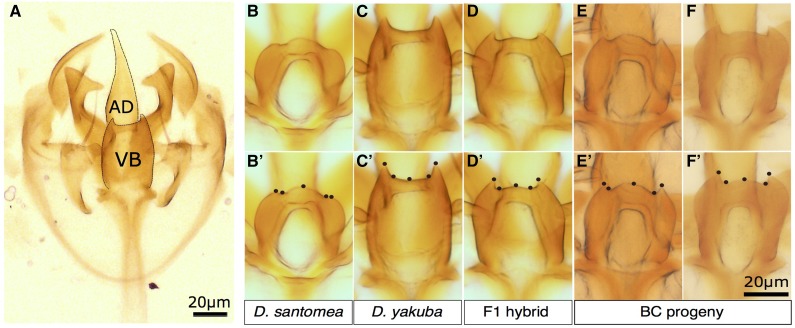
Variation in male ventral branch shape between *D. yakuba*, *D. santomea*, and their hybrid progeny. (A) Dissected *D. yakuba* internal genitalia including the fly’s copulating organ (aedeagus, AD) and the ventral branches (VB). The outline of the ventral branches and of the aedaegus are indicated by a black line. Light microscopy images of the dissected ventral branches in *D. santomea* (B), *D. yakuba* (C), a F1 hybrid male (D), and two backcross progeny individuals (E and F). Corresponding landmark configurations are shown in bottom panels (B′, C′, D′, E′, F′).

Recent QTL mapping studies have greatly benefited from the development of next-generation sequencing technologies. One such technique, multiplexed shotgun genotyping (MSG), is a rapid and low-cost method for genotyping hundreds of individuals at multiple genomic loci using as little as 10 ng of DNA (Andolfatto *et al.* 2011) compared to 1 µg for the restriction-site associated DNA (RAD) approach of Baird *et al.* (2008) and 100 ng for double digest RAD (Peterson *et al.* 2012). MSG infers, through a Hidden Markov Model, ancestry of chromosomal regions using DNA sequences distributed randomly throughout the genome in each individual. In contrast, RAD relies on the sequencing of the same sites in all individuals. We report in this article a new protocol that allows preparation of MSG libraries in approximately one-third the time of the original protocol.

Here we characterized the genetic basis of the shape divergence in male ventral branches between *D. yakuba* and *D. santomea* using F1 hybrids and QTL mapping in a backcross population. We used landmark-based geometric morphometrics to measure the shape of the ventral branches. We identified three loci, accounting for 29%, 14%, and 9% of the main shape difference between species.

## Materials and Methods

### Fly stocks

The *D. yakuba yellow*[1] line #14021-0261.05 was obtained from the San Diego Species Stock Center. The *D. santomea* SYN2005 strain was made by mixing six isofemale lines collected by Jerry A. Coyne in the zone of sympatry with *D. yakuba* in January 2005 (Matute *et al.* 2009). To confirm that these strains produce genitalia with ventral branches typical of each species, we collected *D. santomea* and *D. yakuba* females from São Tomé Island in February 2015 and observed that the first- or second-generation male progeny from these females had genitalia that were qualitatively similar to the strains used for genetic mapping. All flies were cultured on standard cornmeal–agar medium at 25° in uncrowded conditions.

### Parental strains and crosses

*D. yakuba yellow*[1] and *D. santomea* SYN2005 males that were less than 3 days old were collected for genitalia dissection after being reared for at least two generations at 25° in uncrowded conditions. *D. yakuba yellow*[1] virgin females were crossed *en masse* to *D. santomea* SYN2005 males to generate F1 hybrids. F1 hybrid females were subsequently mated to *D. santomea* SYN2005 males to generate backcross males used in QTL mapping. Individual flies were frozen at −80° for a week to a year before dissection.

### Phenotyping

To optimize mounting and obtain reliable landmark configurations, male genitalia were dissected in 1× PBS and positioned wet on the glass slide and slightly air-dried so that they could stick to the slide before adding DMHF (dimethyl hydantoin formaldehyde) mounting medium (available at Entomopraxis) and a cover slip. We then kept only those dissections where the entire internal genitalia were mounted and where the plane of the ventral branches was parallel to the slide. Pictures centered on the ventral branches were taken at 400× magnification with an Olympus IX83 research inverted microscope or a Zeiss Axio Observer.Z1 inverted microscope.

### Morphometric analyses

For each male, landmarks were manually positioned on the pictures of the ventral branches (Figure 1, B′–F′) using the software tpsDIG2 version 2.1 (Rohlf 2006) and compiled in a .tps file as a set of five *x*,*y* coordinates (forming one configuration). With the full set of configurations (Supporting Information, File S1), we performed a full generalized Procrustes analysis (GPA) using the function “procGPA” of the R package “shapes” (version 1.1-9) written by I. L. Dryden and available on the CRAN (http://cran.r-project.org). This function performs an optimized superimposition of the configurations using translation, rotation, and scaling and then a principal component analysis on the Procrustes tangent space coordinates (Dryden and Mardia 1998). The generalized Procrustes analysis was carried out on the backcross progeny dataset only (*n* = 365) or on the full dataset (*n* = 507) comprising parents (*n* = 48 *D. santomea*, 46 *D. yakuba*), F1 hybrids (*n* = 48), and BC progeny (*n* = 365). Resulting files are available as supplementary data (File S2, File S3, File S4). Spine thrust—that is, how much spines are elevated above the middle prominence of the ventral branches—is computed as the maximum of the *y* coordinate for landmarks 1 and 5 minus the *y* coordinate of landmark 3, when the *x*-axis is defined as the axis passing by landmarks 2 and 4 and oriented from 2 to 4, and with the *y* axis defined so that (*x,y*) is an oriented orthonormal basis (Figure S1, File S5).

### Measurement precision

Repeatability of landmark configuration acquisition (*i.e.*, measurement error) was assessed as follows: genitalia of 22 dissected *D. santomea* SYN2005 individuals were mounted on slides five times and photographed. Landmark acquisition was performed on the five distinct images for each individual. All configurations (File S6) were superimposed using a generalized Procrustes analysis (as detailed above). The resulting tangent coordinates (Figure S2) of the 22 individuals × 5 sessions were analyzed using the procD.lm function from the R “geomorph” package version 2.1.2 (Adams and Otarola-Castillo 2013). Briefly, this function performs a Procrustes ANOVA (Goodall 1991; Klingenberg and McIntyre 1998) over the coordinates from each configuration and quantifies the amount of variation attributable to the individual and the session factors in the following linear model: tangent coordinates ∼ individual + session.

### Genotyping

After removal of the genitalia, the body of each male was placed in a 1.5-ml Eppendorf tube and crushed with a manual pestle in 180 μl of Qiagen Tissue Lysis buffer. DNA was extracted using a Qiagen Dneasy Blood & Tissue extraction kit (cat# 69506). After extraction, DNA was kept at −80° before proceeding with genotyping. For genotyping we used a new protocol (Figure S3, File S7) to prepare multiplexed shotgun genotyping (MSG) libraries. The original method described in Andolfatto *et al.* (2011) for making MSG libraries involves fragmentation of genomic DNA with restriction enzymes and ligation of linkers containing inline indexes to each sample. This method is reliable but involves many steps and takes several days to generate a library. Our new method, called Whole Genome Amplification using Manta Polymerase with Degenerate Primers (WMD), allows preparation of libraries from many low mass DNA samples quickly and with fewer steps. In the first step, a nicking endonuclease is used to generate single-stranded nicks in the DNA template (Figure S3). These nicks generate 3′ termini that are suitable substrates for DNA polymerases possessing strong processivity and strand displacement activity, such as Bst polymerase (large fragment), phi29 polymerase, or Sequenase 2.0 (Joneja and Huang 2011). We use the Bst polymerase (large fragment) provided by Enzymatics called Manta polymerase. In an isothermal reaction, the single-stranded DNA generated by the strand-displacement activity of Manta polymerase serves as a template for oligonucleotides containing, from 5′ to 3′: an adaptor sequence, an index of variable length, and a 3′ 12-bp partially degenerate sequence (Figure S3a). This primer contains a 5′ 18-atom hexa-ethyleneglycol spacer to prevent self-priming (Brukner *et al.* 2005). The 3′ partially degenerate sequences serve as new primers for the Manta polymerase. Because multiple oligonucleotides can prime the same strand of DNA, polymerization events located 5′ will displace primed sequences located 3′. These freed single-stranded fragments then serve as templates for additional primers. DNA synthesis from these primers ultimately generates fragments containing primer sequences at both ends, which provide the templates for library preparation with later PCR steps using the adaptor sequences as priming sites (Figure S3b). In the second step of WMD-MSG, samples with different indexes introduced during the initial step are pooled and subjected to polymerase chain reaction with oligonucleotides containing, from 5′ to 3′: a sequence compatible with the adaptors for one of several DNA next-generation sequencing platforms, a second DNA index, and a sequence complementary to the adaptors introduced in the first step (Figure S3c). This dual indexing protocol can provide extremely high levels of multiplexing. Sometimes, WMD-MSG leads to unacceptably high dropout of samples when very large numbers of samples (>500) are processed in a single library. Future optimization of the WMD-MSG protocol may allow improved normalization of read counts among samples, although the current performance of the protocol was sufficient for our study of 384 individuals. A detailed protocol for WMD-MSG is provided as Supplementary Material.

The WMD-MSG library was sequenced on an Illumina HiSeq 2500 and the fastq sequence file was analyzed directly with the MSG software, which has been updated to parse fastq files that contain WMD-MSG barcodes (github.com/JaneliaSciComp/msg). In total, 189,819,468 reads were collected for 384 individuals, among which 365 had reliable phenotypic data. The two species parental genomes used in MSG were prepared by reducing the level of intraspecific polymorphism by applying the script DisambiguateGenomes.py (github.com/dstern/DisambiguateGenomes) sequentially to genomes updated with whole genome sequencing Illumina reads from three strains of *D. yakuba* and three strains of *D. santomea*. The *D. yakuba* reference genome that we updated is dyak-all-chromome-r1.3 available from FTP at Flybase (strain Tai18E2). For *D. yakuba*, we used reads from *D. yakuba* Ivory Coast (San Diego Species Stock Center #14021-0261.00; data from Cande *et al.* 2012), *D. yakuba yellow*[1] (San Diego Species Stock Center #14021-0261.05, sequenced by BGI; this study), and *D. yakuba white*[1] (San Diego Species Stock Center #14021-0261.02). For *D. santomea*, we used reads from *D. santomea* STO-LAGO1482 (collected by Daniel Lachaise in 2001 on São Tomé Island at altitude 1482), *D. santomea* STO.4 (San Diego Species Stock Center #14021-0271.00; data from Cande *et al.* 2012), and *D. santomea* SYN2005 (sequenced by BGI; this study). The resultant posterior probabilities of ancestry (“soft genotypes”) were thinned to informative markers using the script pull_thin_tsv.py (https://github.com/dstern/pull_thin) (File S8, File S9). Markers were considered informative when the conditional probability of being homozygous differed by more than 0.05 from their neighboring markers.

### QTL mapping

QTL mapping was performed using R/qtl (Broman *et al.* 2003; Broman and Sen 2009) (File S10) package version 1.32-10. Briefly, the principle of QTL mapping is to correlate segregating genetic markers with trait values to identify chromosome regions that significantly affect the phenotype of interest, here the shape of the ventral branches, when substituting a *D. santomea* allele for a *D. yakuba* allele. Posterior genotype probabilities, estimated directly by the Hidden Markov Model implemented in MSG, were imported into R-qtl as a “cross” object using the R function read.cross.msg (https://github.com/dstern/read_cross_msg). In our *D. santomea* backcross population we did not find significant departure from the expected distribution of 50% of *D. santomea* alleles and 50% of *D. yakuba* alleles at each marker position. For all traits (BC-PC1, All-PC1, centroid size, spine thrust, see *Results and Discussion* for details), we performed genome scans with a single QTL model (“scanone”) using the Haley-Knott regression method (Haley and Knott 1992), which performs well with datasets having high genotype completion (Broman and Sen 2009). We determined genome-wide statistical significance using 10,000 permutations (Churchill and Doerge 1994). Two QTL peaks above the 1% significance threshold were detected. Both QTL were incorporated into a multiple QTL model using the “fitql” function and we tested for possible interactions between loci. To refine the position of the QTL in the multiple-QTL model, we used the function “refineqtl”, which uses an iterative algorithm to scan neighboring sites for higher LOD (logarithm of odds) score values, taking into account the already found loci (Broman and Sen 2009). Having obtained a first refined model with two QTL, we used the function “addqtl” to detect additional QTL. A third QTL was thus found and introduced in a new multiple model, refined and fitted to account for interactions. Based on the full three-QTL model, we did not find any significant additional QTL with the function “addqtl”: the highest LOD score for a fourth QTL reached only 2.35. We computed the LOD score of the full three-QTL-model, the percentage of variance explained by genetic variation at each locus, and estimated effects of each locus (Broman and Sen 2009). The 2-LOD intervals were calculated using the “lodint” function with parameter drop of 2. QTL effects are defined as the effect caused by the replacement of a *D. santomea* allele by a *D. yakuba* allele. These effects are given either as absolute values, which is the increase in BC-PC1 values, or as a percentage of the parental species difference, which is as a percentage of the species difference for X-linked QTL and as a percentage of half of the species difference for autosomal QTL (as in Macdonald and Goldstein 1999). To calculate the relative effect, the BC-PC1 absolute effect value was converted into an All-PC1 effect value using the following linear model: All-PC1 effect = 1.143 * BC-PC1 effect − 0.62 (formula of the correlation slope between BC-PC1 and All-PC1) (Figure S4); the parental difference was calculated as the difference in means of All-PC1.

### Data availability

All reagents and strains are available on request. Supplementary files, including genotypic and phenotypic data, are available for download in the *G3* journal supporting information section associated with this article. File S1 contains the *x*,*y* coordinates of the landmark configurations of all individuals (*D. santomea*, *D. yakuba*, F1 hybrids, and *D. santomea* backcross progeny). File S2, File S3, File S4, and File S5 contain the All-PC1 scores, BC-PC1 scores, centroid size, and “spine thrust” measures used for QTL mapping. File S6 contains the *x*,*y* coordinates of the landmark configurations of the 22 *D. santomea* individuals assessed for measurement precision. The WMD-MSG detailed protocol including primer sequences is available as a PDF in File S7. File S8 contains ancestry estimates for the *D. santomea* backcross after application of the Hidden Markov Model. File S9 contains ancestry estimates for the *D. santomea* backcross after application of the Hidden Markov Model and thinning to include only neighboring markers whose conditional probability differed by at least 0.05. The QTL mapping script is provided in .R format in File S10.

## Results and Discussion

The most conspicuous difference in genital morphology that has been reported between *D. yakuba* and *D. santomea* is the shape of the ventral branches that cover the aedeagus (Kamimura and Mitsumoto 2012a; Kamimura 2012; Yassin and Orgogozo 2013). We examined the ventral branches shape in two lines, *D. yakuba yellow*[1] and *D. santomea* SYN2005. As previously described for other strains (Kamimura and Mitsumoto 2012a; Kamimura 2012; Yassin and Orgogozo 2013), we found spiny ventral branches in *D. yakuba* and no such spines in *D. santomea* males (Figures 1, A–C). To identify the genetic loci underlying this genital difference, we developed a method to measure the shape of the ventral branches in a quantitative manner and used it for QTL mapping in a *D. santomea* backcross population.

### A reliable quantitative measure for ventral branches shape

Previous genetics studies of genitalia shape have quantified shape variation using elliptic Fourier analysis, which relies on organ outlines (Kuhl and Giardina 1982; Ferson *et al.* 1985) and decomposes contours into a series of harmonics that can be described by their coefficients, the Fourier coefficients. In our case, the point at which the ventral branches emerge from the aedeagus was difficult to locate reliably, making it impractical to define the ventral branch contour and to use outline-based methods of shape decomposition, such as elliptic Fourier analysis. Therefore, we used a geometric morphometric approach based on landmarks. On the ventral branches we found only five landmarks that were unambiguously comparable between males; these corresponded to ventral branch contour discontinuities at the distal end of the genital structure. We used these five landmarks to quantify precisely and accurately the shape of the ventral branches (Figure 1). These landmarks, although not documenting the shape of the ventral branches at their base, where they emerge from the aedeagus, allowed us to describe and quantify the shape of the distal end of the ventral branches, and especially the relative length and width of the spines to identify the main axes of shape variation in our QTL mapping population.

For each individual we manually positioned a series of five landmarks, forming one configuration (Figure 1, B′–F′). We then tested whether landmark acquisition, and therefore shape characterization, was robust to the mounting and acquisition process. Five sessions of mounting and unmounting of internal genitalia followed by landmark acquisition for the same 22 *D. santomea* individuals showed no effect of the session on the landmark configuration (F = 3.38, *P* > 0.05, Procrustes-ANOVA) and yielded very similar configurations (Figure S2). We concluded that our quantitative measure of ventral branches shape is reproducible and appropriate for QTL mapping.

### The main axis of variation in the backcross matches the interspecific difference

We crossed *D. yakuba* females with *D. santomea* males to generate F1 hybrid males. In these hybrids the spine shapes were qualitatively more similar to *D. yakuba* than to *D. santomea* (Figure 1, B–D), suggesting that *D. yakuba* alleles display stronger dominance for spine shape. Therefore, we decided to perform a QTL mapping on a *D. santomea* backcross population, rather than on a *D. yakuba* backcross population or on a combination of both backcrosses. We analyzed individual landmark configurations from *D. santomea* parents, *D. yakuba* parents, F1 hybrids, and backcross progeny altogether by performing a Procrustes superimposition (Gower 1975; Rohlf and Slice 1990).

**Figure 2:**
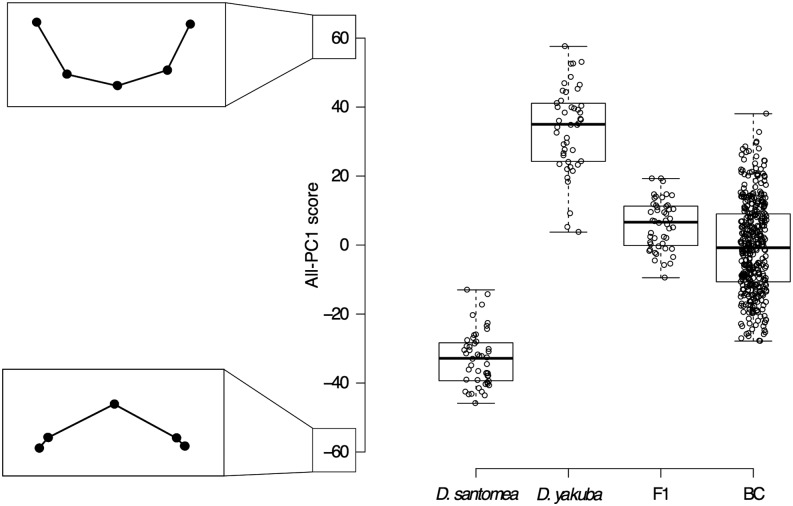
Distribution of All-PC1 scores in parental species, F1 hybrids, and backcross progeny. The *y*-axis represents the first principal component (All-PC1) of the Generalized Procrustes analysis on the full dataset. Individual All-PC1 scores (dots), median (thick line), first and third quartiles (box limits), and minimum and maximum All-PC1 scores (notches) are represented for each genotype. Characteristic configurations, for which All-PC1 = −60 (bottom) or All-PC1 = 60 (top) and for which other All-PCs = 0, are shown on the left side of the *y*-axis and depict variation along All-PC1 alone.

From the superimposition of all configurations, we performed a principal component analysis (PCA) (Dryden and Mardia 1998) to identify the main axes of morphological variation in our dataset. This analysis identified one main principal component, which we named All-PC1 and which described 57.8% of the variation in our dataset. The next three principal components explained approximately 10% of the variance each (Figure S5). Shape variation along All-PC1 matches the interspecific shape difference: at one extreme of the All-PC1 axis, ventral branches shape are rounder and at the other extreme they are spinier. More precisely, as we move along All-PC1, shape changes from rounded to spinier ventral branches through the flattening of the central ridge (landmark 3) together with the rise of landmarks 1 and 5 above that ridge (Figure 2). The distributions of All-PC1 scores for *D. santomea* and *D. yakuba* individuals do not overlap (Figure 2). Altogether, these observations make All-PC1 a good quantifier of shape variation that is relevant for the study of the interspecific difference. Overlapping of F1 hybrids with *D. yakuba* All-PC1 scores but not with *D. santomea* All-PC1 scores (Figure 2) confirms that F1 hybrids are more similar in spine shape to *D. yakuba* than to *D. santomea*. *D. santomea* backcross progeny produced genitalia with All-PC1 scores that ranged approximately from the mean for *D. yakuba* to the mean for *D. santomea* parents (Figure 2).

**Figure 3:**
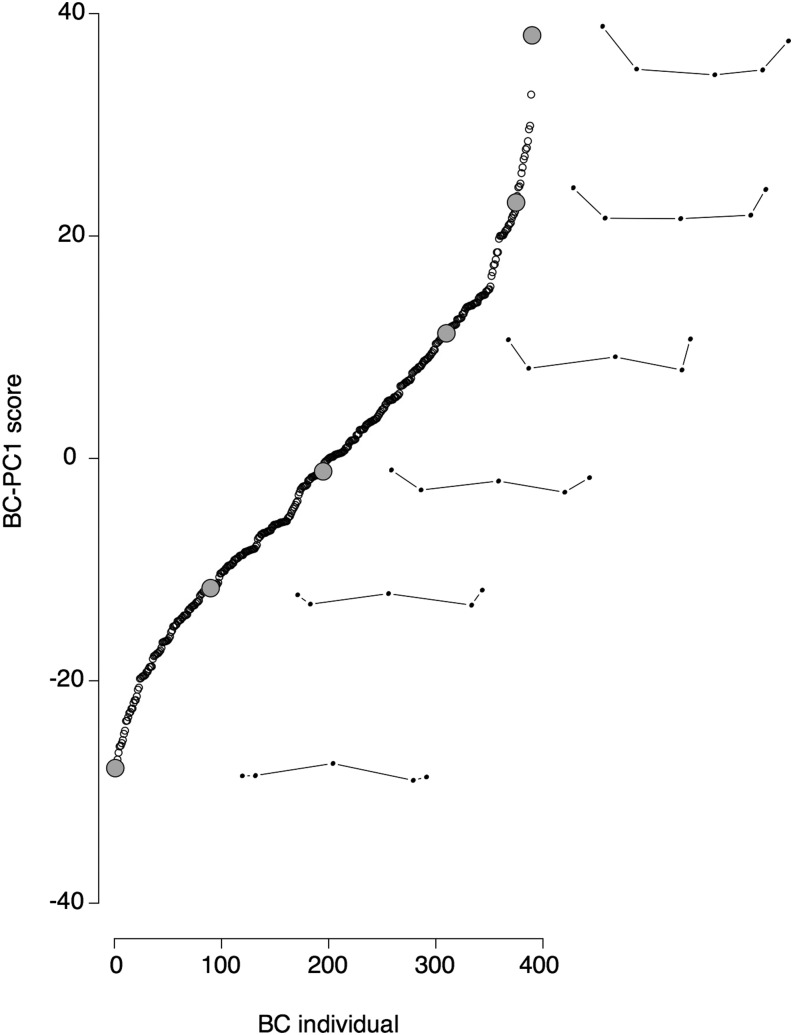
Distribution of BC-PC1 scores in the backcross progeny and actual landmark configurations along the BC-PC1 axis. Distribution of all the backcross progeny individuals is shown along the first principal component of a Generalized Procrustes analysis based on the backcross progeny only (BC-PC1). For individuals #1, #90, #195, #310, #375, and #390 (large gray dots), with respective scores of −27.8, −11. 7, −1.2, 11.3, 23, and 38, the actual configuration is shown on the right of the gray dot.

To test whether shape variation within the backcross progeny alone is different from the overall shape variation including the backcross, parental strains, and F1 hybrids, we performed the morphometric analysis on the configurations of the backcross progeny only. We identified one main principal component, which we named BC-PC1 and which explains 40.9% of the variation (Figure S3). BC-PC1 scores recapitulate the interspecific pattern of variation (Figure 3) and are highly correlated with All-PC1 scores (r^2^ = 0.996) (Figure S4). This indicates that the main axis of shape variation for the backcross matches well the interspecific difference between *D. yakuba* and *D. santomea*.

In most morphometric QTL studies, phenotypic variation is distributed among multiple principal components, suggesting that individual QTL would have mainly small effects and alter the phenotype in directions that do not necessarily match the shape variation between parental strains. In our study we identified a major principal component that explains a relatively large part of the shape variation in our QTL mapping population, which is rare in genitalia evolution QTL studies (Liu *et al.* 1996; McNeil *et al.* 2011; Takahara and Takahashi 2015).

In conclusion, we found one major principal component, BC-PC1, which explains most of the shape variation in the backcross progeny and which corresponds to the interspecific spine shape difference between *D. yakuba* and *D. santomea*.

### A new method for genotyping a high number of markers rapidly and efficiently

High-throughput sequencing libraries were prepared from single flies and low concentration DNA, with little hands-on time in less than 1 days, using an improved method of multiplexed shotgun genotyping (MSG), which we report here. This new procedure, whole genome amplification using Manta polymerase with degenerate primers (WMD-MSG), relies on the strength of the previously published MSG genotyping technique (Andolfatto *et al.* 2011) and introduces a fast and efficient library preparation protocol that relies on a nicking enzyme followed by amplification with a strand displacement activity polymerase (Figure S3). The two-step library preparation protocol uses two sets of barcodes, allowing high multiplexing with a small number of primers. In a matter of a few days, we genotyped 384 backcross progeny individuals and obtained a total of 511,234 markers genome-wide, including 4499 informative markers.

### One narrow locus on chromosome 3L explains interspecific shape variation

**Figure 4:**
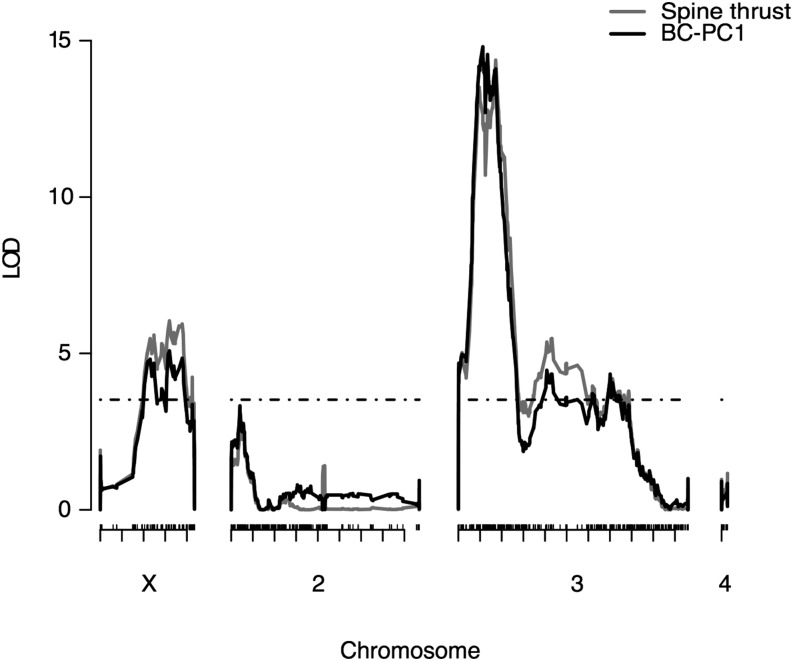
QTL analysis of ventral branch shape in the *D. santomea* backcross. On the *y*-axis are the LOD profiles from a Haley-Knott regression analysis for BC-PC1 (solid black line) and for “spine thrust” (see text for details). The *x*-axis represents the physical map based on the *D. yakuba* genome. Distance between two ticks below the *x*-axis represent 0.5 Mb. Ticks above the *x*-axis represent informative markers from WMD-MSG. Note that few informative markers are found on the right arm of chromosome 2, indicating that there is at least one inversion between our parental lines. The dotted line represents the 1% significance threshold (similar for both phenotype values).

An initial genome scan for ventral branch shape (BC-PC1) yielded two significant QTL, one on chromosome X and one on chromosome 3 (Figure 4). To check for additional QTL, we built a multiple QTL model with these two QTL and scanned for additional QTL taking these two QTL into account (see *Materials and Methods*). Having found an additional QTL on chromosome 2, we built a refined multiple-QTL model and checked for epistatic interactions between these three QTL (Broman and Sen 2009). This three-QTL model gave a LOD score of 25.5 and explained 28% of the variance in our backcross population (Table 1). A search for a fourth QTL did not detect any other significant QTL. Our analysis did not reveal any epistatic interactions between the three loci. In summary, QTL mapping of BC-PC1 in the backcross progeny revealed three QTL: one narrow LOD peak on chromosome 3L with a maximum LOD score of 17.0, another locus on chromosome 2L (LOD = 4.9), and a wider region on chromosome X (LOD = 6.14) (Table 1). The LOD scores for all three peaks exceeded a statistical significance threshold of *P* < 0.01. QTL mapping of All-PC1 yielded the same three QTL (Figure S6).

**Table 1:** Multiple QTL model for BC-PC1

	QTL1	QTL2	QTL3
QTL locations	chrX:15,971,231	chr2:2,115,180	chr3:8,387,508
Effect sizes:			
Absolute	5.54	4.48	8.88
Relative to species difference	8.6%	13.5%	28.6%
LOD Drop one*^a^*	6.7	4.9	17.0
2-LOD interval	chrX:9,749,735.. 19,881,209	chr2:583,024..3, 736,003	chr3:6,415,420.. 9,089,467
Interval physical size	10.13 Mb	3.15 Mb	2.67 Mb
Number of predicted genes*^b^*	1137	445	338

Results from a 3-QTL model are shown. The 3-QTL model has a LOD score of 25.5 and explains 28% of the variance.

a Reduction in the LOD score of the full model when this QTL was removed.

b Number of predicted genes from the annotation of the *D. yakuba* (R1.04) genome.

The shape difference that we have mapped could be linked to a difference in ventral branch size. For example, individuals with a *D. yakuba*–like spiny shape could have bigger ventral branches than individuals with a *D. santomea*–like rounded shape. In this case, the species difference would mainly reflect a difference in organ size rather than shape. In our study, we find that centroid size of ventral branches configurations is not correlated with BC-PC1 (r^2^ = 0.06) (Figure S7) and does not map to any QTL (Figure S8). This suggests that the QTL we identified with BC-PC1 and All-PC1 are QTL of shape and not size.

**Figure 5:**
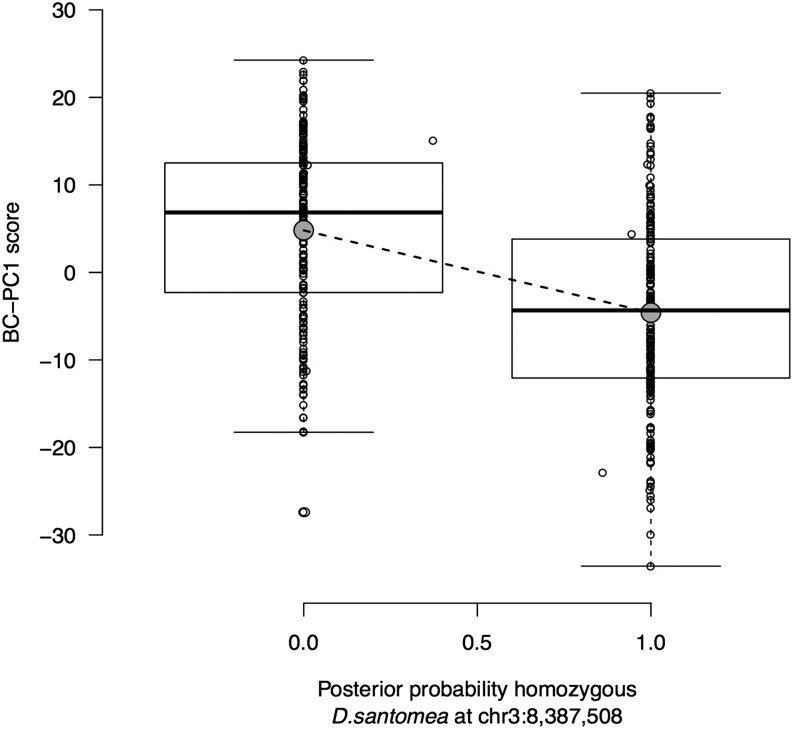
Effect plot of the main QTL on chromosome 3 for the BC-PC1 scores. The BC-PC1 score is indicated as a function of the posterior probability of homozygosity at position chr3:8,387,508 for the *D. santomea* backcross progeny males. Individual BC-PC1 scores (dots, *n* = 365), median (thick black line), first and third quartiles (box limits), minimum and maximum BC-PC1 scores (notches), and means (gray dots) are shown. The dotted line indicates the linear model of BC-PC1 score as a function of the genotype.

Based on 2-LOD intervals, the major QTL on chromosome 3 corresponds to a small region of 2.67 Mb with an estimated number of 338 genes in the *D. yakuba* genome. This major QTL has the strongest effect on the variation in shape in the backcross population. We find that the mean BC-PC1 score of heterozygous individuals is increased by 8.88 (28.6% of the mean parental species difference), compared to homozygous individuals from the mapping population, implying that substitution of one *D. santomea* allele for a *D. yakuba* allele, at this locus, increases the BC-PC1 score by one-third of the species difference in shape quantified by our morphometric analysis (Figure 5, Table 1). The QTL on chromosome X covers a region of 10 Mb including more than 1000 genes and the QTL on chromosome 2 covers 3.15 Mb with a predicted number of 445 genes in the *D. yakuba* genome (Table 1). Both of these QTL have smaller phenotypic effects (8.6% and 13.5% of the parental species difference, respectively) than the major QTL on chromosome 3.

### A simple measure of spine length that relates to the lock-and-key mechanism leads to the same QTL

As pointed out in a previous study of male genitalia in *D. melanogaster* (McNeil *et al.* 2011), principal components are specific to a given dataset because they describe the axes of variation of that particular dataset, and therefore it can be difficult to relate principal components to familiar shapes. Visual inspection of landmark configurations along the BC-PC1 axis (Figure 3) suggested that BC-PC1 quantifies how much the spines are elevated above the middle prominence of the ventral branches. We derived a simple measure, which we called “spine thrust” and which captures the maximum spine length relative to the middle prominence (see *Materials and Methods* and Figure S1). We found that spine thrust is highly correlated to BC-PC1 (Figure S9), but not to centroid size (Figure S10). QTL mapping for “spine thrust” yielded the same QTL as BC-PC1 (Figure 4). “Spine thrust” may relate to how harmful the ventral branches are for the *D. santomea* female (Kamimura 2012). If spines do not extend beyond the middle prominence of the structure, then they should not damage the female. In contrast, higher “spine thrust” is expected to be associated with deeper wounds.

We found that all three QTL effects act in the same direction, that is, spine thrust (as well as BC-PC1 and All-PC1) increases when a *D. santomea* allele is substituted by a *D. yakuba* allele at both loci (Table 1), an argument in support of directional selection for spine thrust divergence (Orr 1998). Because spine shape has changed dramatically between species of the *yakuba* complex, it is currently impossible to infer with certainty the direction of the evolutionary changes between *D. yakuba* and *D. santomea*. The most closely related species, *D. teissieri*, has spines that are finer and longer than those of *D. yakuba* (Yassin and Orgogozo 2013; Kamimura and Mitsumoto 2012a,b), and other species of the *D. melanogaster* subgroup are devoid of spines and ventral branches (Yassin and Orgogozo 2013). Under one scenario, supported by biogeographic data (Lachaise *et al.* 2000), the ancestor of both *D. yakuba* and *D. santomea* might have had ventral branches that looked like *D. yakuba*, and spines may have been lost in the *D. santomea* lineage. Alternatively, the ancestor of both *D. yakuba* and *D. santomea* might have had *D. santomea*–like ventral branches and spines may have evolved in the *D. yakuba* lineage. The identification of the precise nucleotide changes involved in the ventral branches shape difference between *D. yakuba* and *D. santomea* could help to shed further light on the direction of the evolutionary changes.

### Evolution of ventral branches involves at least one locus of relatively large effect

In a previous QTL study of genital shape in *Drosophila*, Zeng *et al.* (2000) identified 19 loci involved in posterior lobe shape evolution between *D. simulans* and *D. mauritiana*, and the largest effect QTL was only 12.4% of the phenotypic difference between the F1 and the backcrossed parental species. By comparison, our QTL study of ventral branches shape identified three QTL whose effects are relatively large: 28.6%, 13.5%, and 8.6% of the parental difference (Table 1). This genetic architecture was found with three independent phenotypic measures (All-PC1, BC-PC1, and “spine thrust”). As highlighted by Cornforth and Long (2003) and by Mackay *et al.* (2009), fine mapping may reveal multiple linked loci of small effects. The three QTL we identified may each contain several genes that are involved in the difference in ventral branches shape between *D. santomea* and *D. yakuba*. Although our results remain to be confirmed by finer mapping with introgression lines, they are currently supported by the high LOD score of our major QTL and by the relatively large number of genotyped individuals in our mapping population.

Our results suggest that the genetic architecture of genitalia morphology divergence may not always be as complex as was discovered for posterior lobe shape. Although most work on *Drosophila* genital evolution had been focused on the posterior lobe, studies of genital trait, and especially those with better understood roles in copulation, will provide a more global view of genitalia evolution.

## 

## Supplementary Material

Supporting Information
