# Supplementary material for: A Major Locus Controls a Genital Shape Difference Involved in Reproductive Isolation Between Drosophila yakuba and Drosophila santomea
Source: G3 (Bethesda). 2015 Oct 27;5(12):2893–901. doi: 10.1534/g3.115.023481 (PMC4683660; doi:10.1534/g3.115.023481)
Supplement: Supporting Information [file supp_5_12_2893__index.html]

A Major Locus Controls a Genital Shape Difference Involved in Reproductive Isolation Between Drosophila yakuba and Drosophila santomea — Supporting Information 

# A Major Locus Controls a Genital Shape Difference Involved in Reproductive Isolation Between *Drosophila yakuba* and *Drosophila santomea*

## Supporting Information for Peluffo *et al.*, 2015

**Files in this Data Supplement:**

- Supporting Information - File contains all supporting figures and legends for supporting files. (.pdf, 1,705 KB)
- Figure S6 - QTL analysis of BC-PC1 and all-PC1 scores in the *D. santomea* backcross. (.pdf, 1,621 KB)
- Figure S7 - Correlation between BC-PC1 and the centroid size of landmark configurations. (.pdf, 1,620 KB)
- Figure S8 - QTL analysis of BC-PC1 and centroid size in the *D. santomea* backcross. (.pdf, 1,621 KB)
- Figure S9 - Correlation between BC-PC1 and "spine thrust". (.pdf, 1,620 KB)
- Figure S10 - Correlation between "spine thrust" and centroid size. (.pdf, 1,620 KB)
- File S7 - WMD-MSG detailed protocol including primer sequences. (.pdf, 493 KB)
- Figure S1 - Illustration of the "spine thrust" measure for a *D. santomea* backcross individual. (.pdf, 1,620 KB)
- Figure S2 - Repeatability of configuration acquisition for *D. santomea* individuals. (.pdf, 1,620 KB)
- Figure S3 - Whole genome amplification using manta polymerase with degenerate primers (WMD). (.pdf, 1,624 KB)
- Figure S4 - Correlation between BC-PC1 and all-PC1. (.pdf, 1,620 KB)
- Figure S5 - Cumulative variance explained by principal component analysis of the generalized Procrustes analysis of ventral branch landmark configurations. (.pdf, 1,621 KB)
- File S1.csv - *x,y* coordinates of the landmark configurations of all individuals (*D. santomea*, *D. yakuba*, F1 hybrids and *D. santomea* backcross progeny). (.csv, 39 KB)
- File S2 - All-PC1 scores phenotype file for QTL mapping. (.csv, 21 KB)
- File S3 - BC-PC1 scores phenotype file for QTL mapping. (.csv, 21 KB)
- File S4 - Centroid size phenotype file for QTL mapping. (.csv, 21 KB)
- File S5 - "Spine thrust" phenotype file for QTL mapping. (.csv, 21 KB)
- File S6 - *x,y* coordinates of the landmark configurations of the 22 *D. santomea* individuals assessed for measurement precision. (.csv, 7 KB)
- File S8 - *D. santomea* backcross genotype sorted data. This file contains ancestry estimates for the *D. santomea* backcross after application of the Hidden Markov Model. (.csv, 4,792 KB)
- File S9 - *D. santomea* backcross genotype pulled thinned data. This file contains ancestry estimates for the *D. santomea* backcross after application of the Hidden Markov Model and thinning to include only neighboring markers whose conditional probability differed by at least 0.05. (.zip, 272 KB)
- File S10 - Qtl mapping script. (.R, 8 KB)
