# Supplementary material for: A Major Locus Controls a Genital Shape Difference Involved in Reproductive Isolation Between Drosophila yakuba and Drosophila santomea
Source: G3 (Bethesda). 2015 Oct 27;5(12):2893–901. doi: 10.1534/g3.115.023481 (PMC4683660; doi:10.1534/g3.115.023481)
Supplement: Supporting Information [file supp_g3.115.023481_023481SI.pdf]

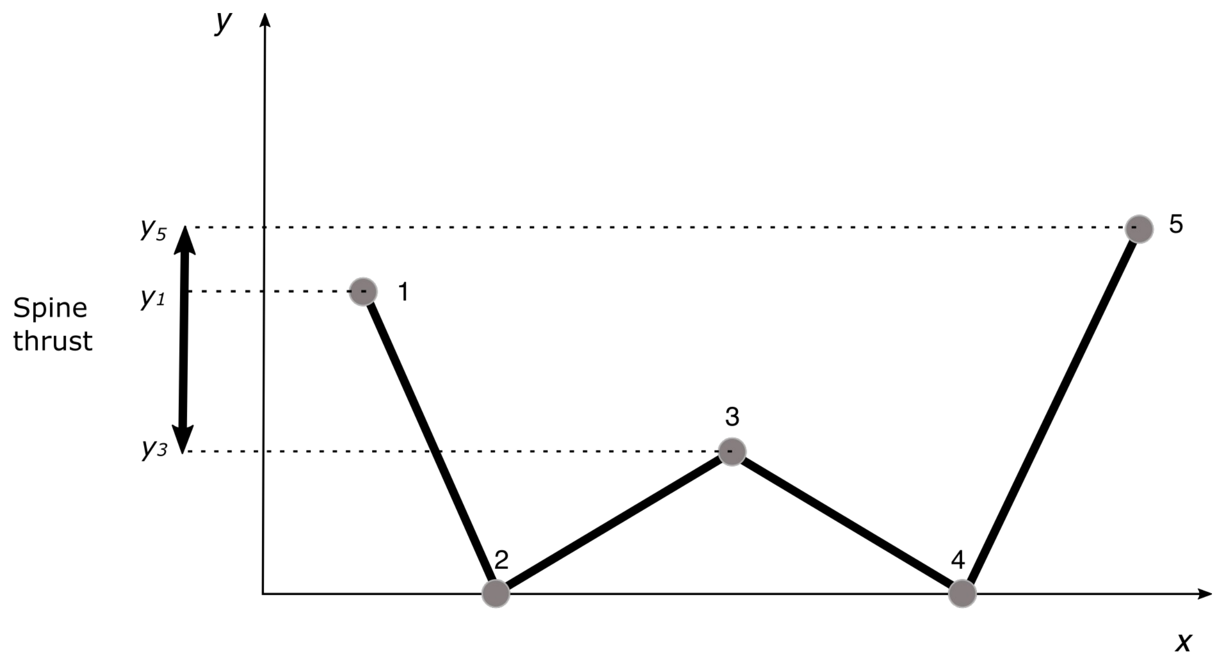

Figure S1. Illustration of the "spine thrust" measure for a *D. santomea* backcross individual. For each individual "spine thrust" is computed as max(*y1*;*y*5)-*y*3 where *y*i is the coordinate of landmark i when the *x*-axis is defined as the axis passing by landmarks 2 and 4, oriented from 2 to 4, and with the *y*-axis defined so that (*x*,*y*) is an oriented orthonormal basis.

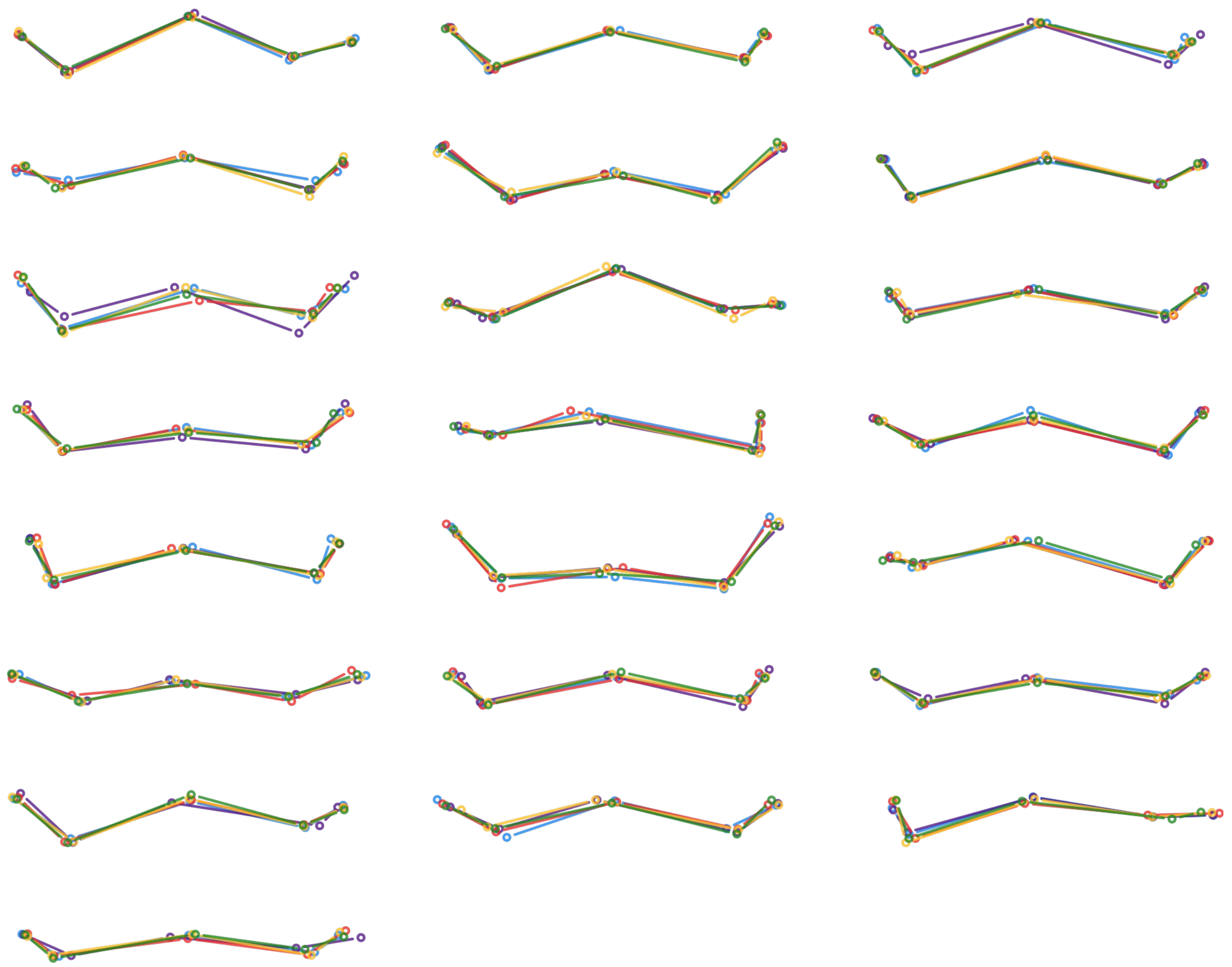

Figure S2. Repeatability of configuration acquisition for *D. santomea* individuals. 22 individuals are shown. For each individual, five sessions of sample mounting, picture acquisition and landmark acquisition were done. Each color represents one session. Configurations where aligned with a generalized Procrustes superimposition for each individual.

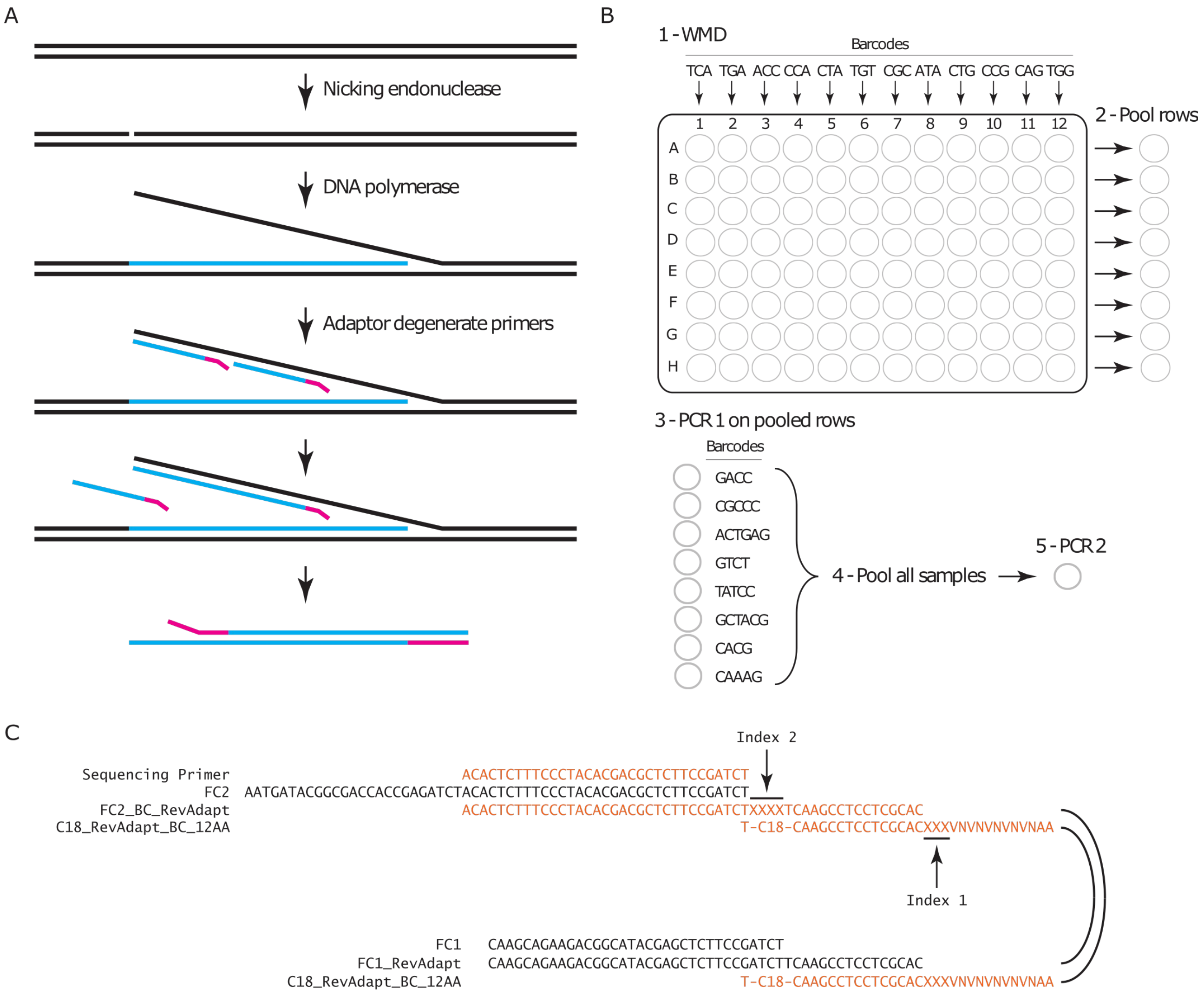

Figure S3. Whole genome amplification using manta polymerase with degenerate primers (WMD). (a) A schematic of the critical steps during WMD. From top to bottom, first, DNA is cleaved site-specifically on a single strand using the engineered restriction enzyme, Nt.Bpu10I. Second, the mesothermic DNA polymerase Manta 1.0 DNA polymerase initiates DNA synthesis from the 3’ end of the nicked DNA (blue strand). Manta polymerase has strong strand displacement activity, which results in the release of a single-strand of DNA. Third, adaptor oligonucleotides containing a partially degenerate 3’ end (pink) hybridize to multiple regions on the single-stranded DNA and provide templates for DNA synthesis (blue) by Manta DNA polymerase. Fourth, the strand displacement activity of Manta polymerase leads to release of single-stranded fragments containing an adaptor oligonucleotide at their 5’ end. Finally, these single-stranded fragments serve as templates for priming by additional adaptor oligonucleotides. Synthesis of the complementary strand produces single stranded products (the top strand of the final product) that are suitable for addition of platform-specific adaptor sequences by polymerase chain reaction. In practice, the three reagents (restriction enzyme, mesothermic DNA polymerase, and modified adaptor oligonucleotides) are combined with DNA and incubated in a single tube, first at 37°C to ensure restriction digestion and then at 50°C to facilitate DNA polymerization. The restriction

enzyme used here, Nt.Npu10I, may continue to cut at 50°C and is inactivated by an 80°C step at the end of the protocol. (b) Workflow for a single plate of 96 samples. First, WMD is performed on samples with a separate barcoded C18_RevAdapt_BC_12AA oligonucleotide used in each column of a 96-well plate. Second, samples are pooled by row. Third, PCR is performed on samples of pooled rows to introduce sequencing platform-specific sequences and a second barcode of variable length. The variable length barcode increases the fidelity of Illumina-based sequencing. Finally, all samples are pooled, cleaned to eliminate residual primers, and a final PCR is performed on the entire sample with platform-specific primers. The sample is now ready for purification, quantification, and sequencing. (c) Diagram illustrating complementarity of the adaptor oligonucleotides containing partially degenerate sequences at their 3' end (pink), and the oligonucleotides used for sequencing on an Illumina platform. The first round of PCR is performed using the FC2_BC_RevAdapt and FC1_RevAdapt oligonucleotides to add a second index sequence to the sequenced end of the fragments. A second round of PCR is performed with FC2 and FC1 oligonucleotides to produce the final library.

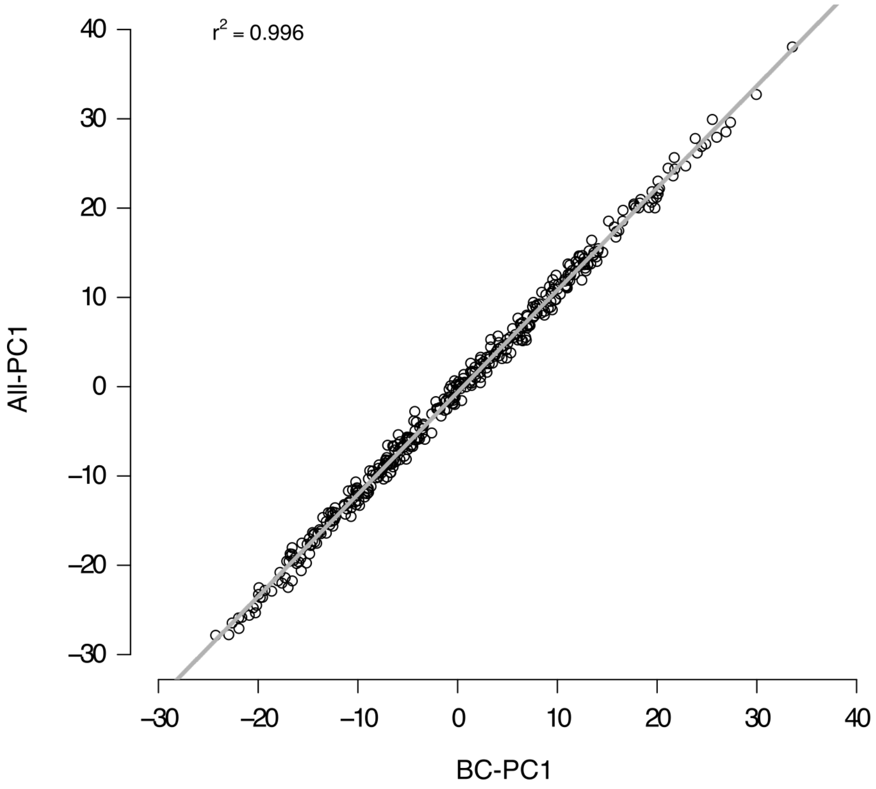

Figure S4. Correlation between BC-PC1 and all-PC1. Each point represents one *D. santomea* backcross progeny individual.

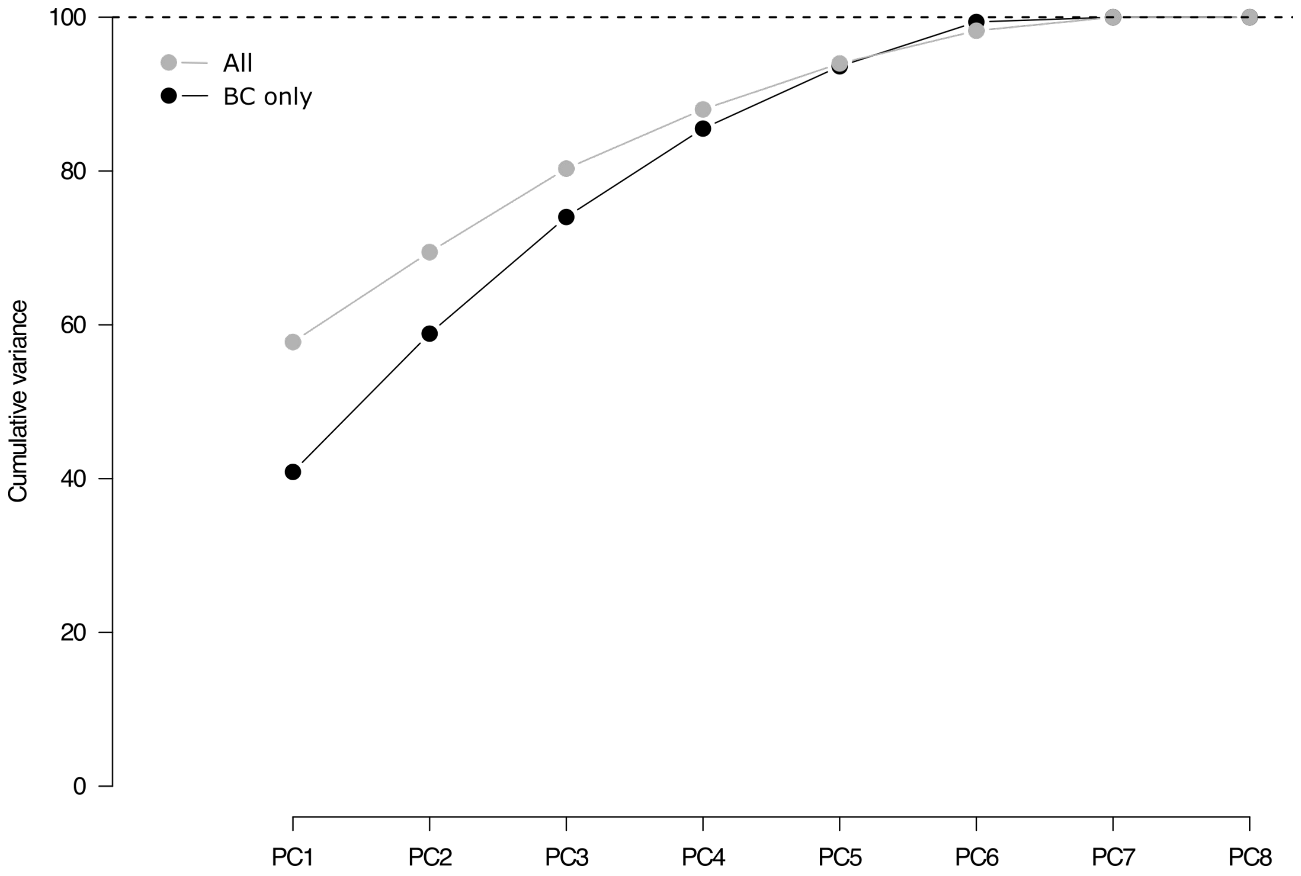

Figure S5. Cumulative variance explained by principal component analysis of the generalized Procrustes analysis of ventral branch landmark configurations. Principal component analysis (PCA) performed only on the backcross progeny (gray) and on the full dataset comprising the two parental species, F1 hybrids and backcross progeny (black) are shown. For the full dataset PCA, the first eight principal components explain 57.8, 11.7, 10.8, 7.7, 6.0, 4.3, 1.8, and 9.1×10^{-10} percent of the variance. For the Backcross PCA, the first eight principal components explain 40.9, 18.0, 15.2, 11.5, 8.0, 5.8, 0.6, and 3.5×10^{-8} percent of the variance.

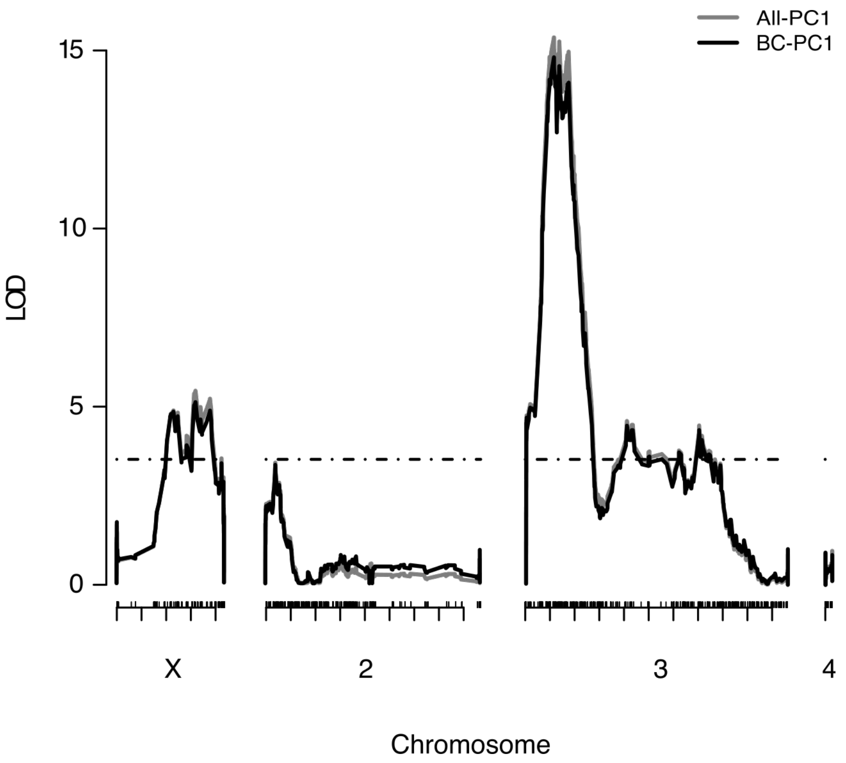

Figure S6. QTL analysis of BC-PC1 and all-PC1 scores in the *D. santomea* backcross. LOD profiles from a Haley-Knott regression analysis for BC-PC1 (generalized Procrustes analysis performed only on the backcross progeny) and for all-PC1 (generalized Procrustes analysis perfomed on the full dataset comprising the two parental species, F1 hybrids and the backcross progeny). The dotted line represents the 1% significance threshold (same for BC-PC1 and all-PC1). Coordinates are given in mega-base pair. Distance between two ticks below the *x*-axis represent 0.5 Mb. Ticks above the *x*-axis represent informative markers from WMD-MSG, i.e. markers flanking recombination events.

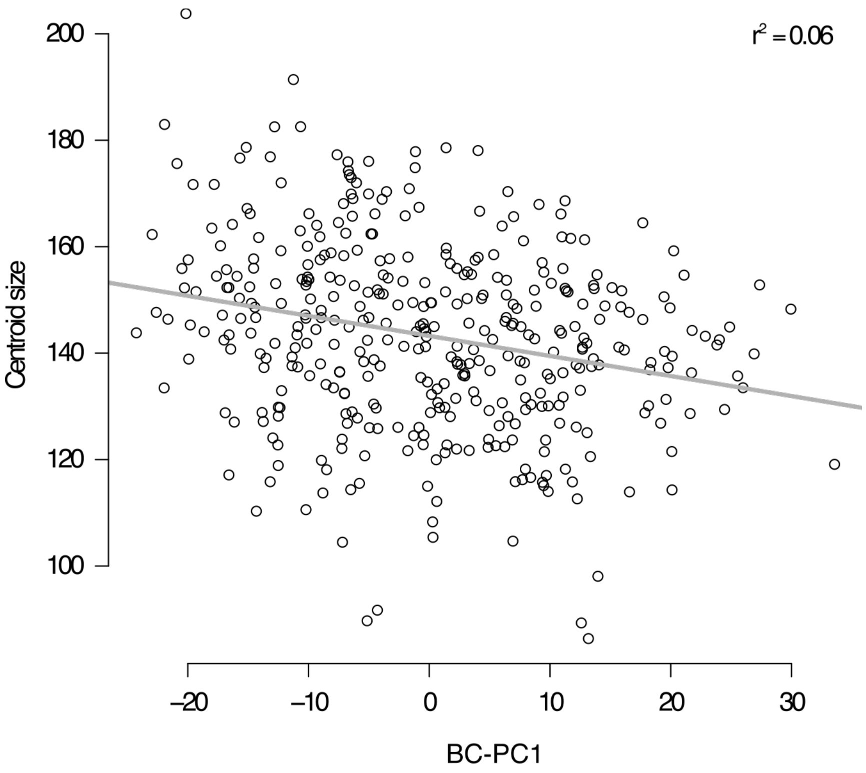

Figure S7. Correlation between BC-PC1 and the centroid size of landmark configurations. Each point represents one *D. santomea* backcross progeny individual.

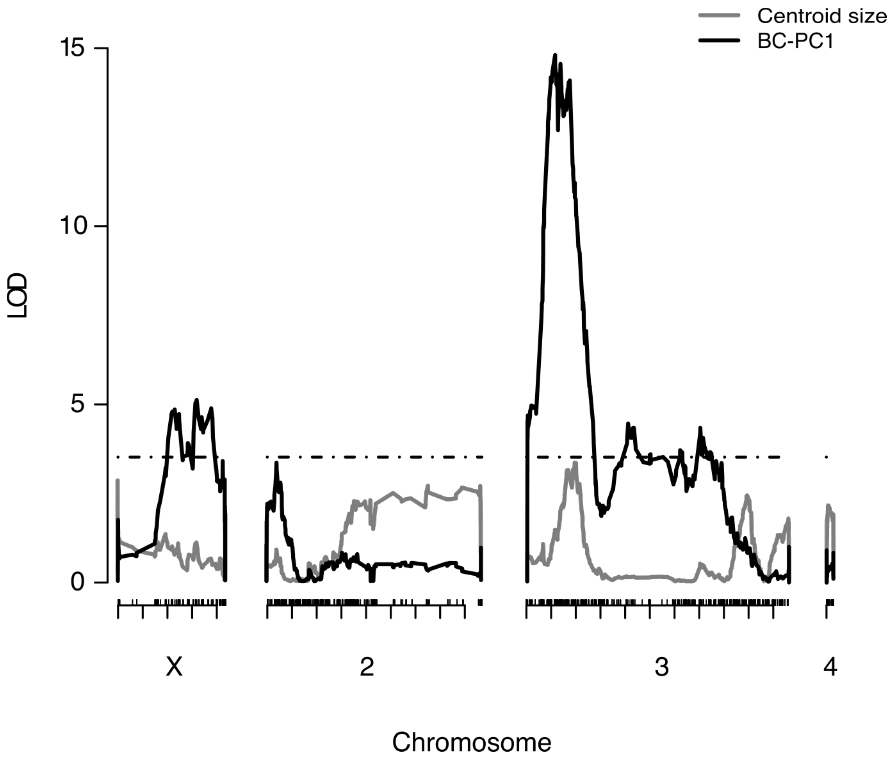

Figure S8. QTL analysis of BC-PC1 and centroid size in the *D. santomea* backcross. LOD profiles from a Haley-Knott regression analysis for BC-PC1 (generalized Procrustes analysis performed only on the backcross progeny) and for centroid size. The dotted line represents the 1% significance threshold (same for BC-PC1 and centroid size). Coordinates are given in mega-base pairs. Distance between two ticks below the *x*-axis represent 0.5 Mb. Ticks above the *x*-axis represent informative markers from WMD-MSG.

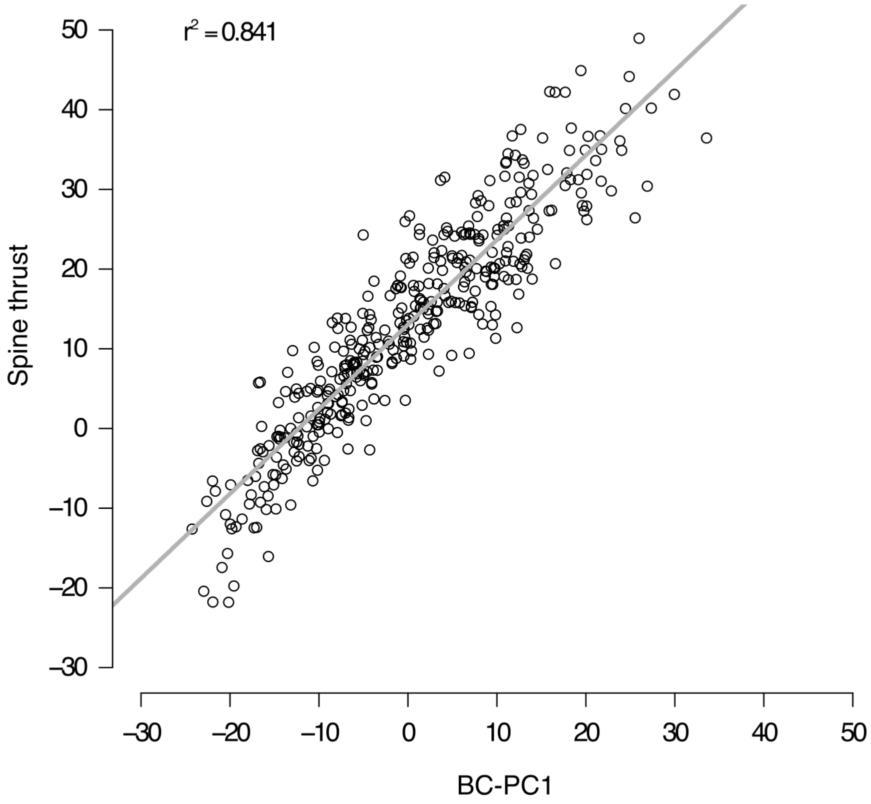

Figure S9. Correlation between BC-PC1 and "spine thrust". Each point represents one *D. santomea* backcross progeny individual.

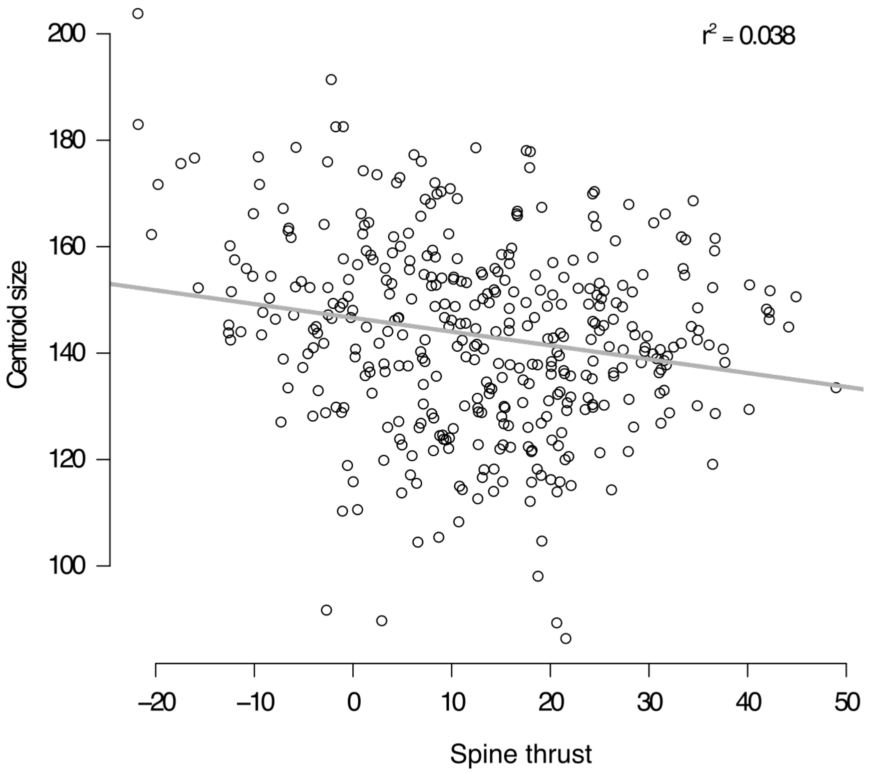

Figure S10. Correlation between "spine thrust" and centroid size. Each point represents one *D. santomea* backcross progeny individual.

File S1-S10

Available for download at: www.g3journal.org/lookup/suppl/doi:10.1534/g3.115.023481/-/DC1

File S1. *x,y* coordinates of the landmark configurations of all individuals (*D. santomea, D. yakuba*, F1 hybrids and *D. santomea* backcross progeny). (CSV)

File S2. All-PC1 scores phenotype file for QTL mapping. (CSV)

File S3. BC-PC1 scores phenotype file for QTL mapping. (CSV)

File S4. Centroid size phenotype file for QTL mapping**.** (CSV)

File S5. "Spine thrust" phenotype file for QTL mapping. (CSV)

File S6. *x,y* coordinates of the landmark configurations of the 22 *D. santomea* individuals assessed for measurement precision. (CSV)

File S7. WMD-MSG detailed protocol including primer sequences. (PDF).

File S8. *D. santomea* backcross genotype sorted data. This file contains ancestry estimates for the *D. santomea* backcross after application of the Hidden Markov Model. (CSV)

File S9. *D. santomea* backcross genotype pulled thinned data. This file contains ancestry estimates for the *D. santomea* backcross after application of the Hidden Markov Model and thinning to include only neighboring markers whose conditional probability differed by at least 0.05. (CSV)

File S10. Qtl mapping script. (R file)
