# Supplementary material for: A Major Locus Controls a Genital Shape Difference Involved in Reproductive Isolation Between Drosophila yakuba and Drosophila santomea
Source: G3 (Bethesda). 2015 Oct 27;5(12):2893–901. doi: 10.1534/g3.115.023481 (PMC4683660; doi:10.1534/g3.115.023481)
Supplement: Supporting Information [file supp_g3.115.023481_FigureS6.pdf]

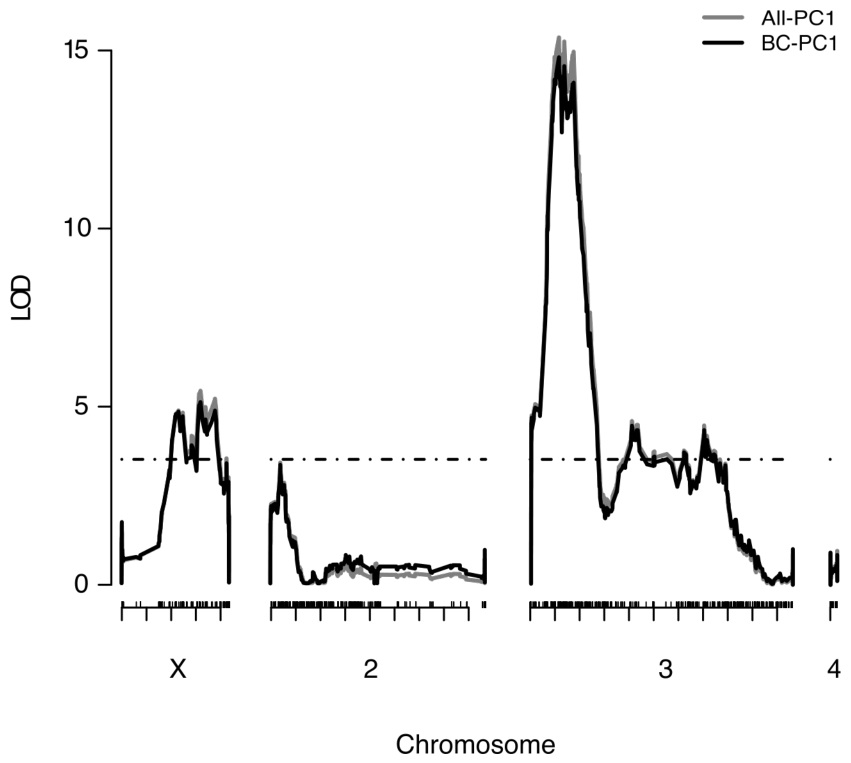

Figure S6. QTL analysis of BC-PC1 and all-PC1 scores in the *D. santomea* backcross. LOD profiles from a Haley-Knott regression analysis for BC-PC1 (generalized Procrustes analysis performed only on the backcross progeny) and for all-PC1 (generalized Procrustes analysis perfomed on the full dataset comprising the two parental species, F1 hybrids and the backcross progeny). The dotted line represents the 1% significance threshold (same for BC-PC1 and all-PC1). Coordinates are given in mega-base pair. Distance between two ticks below the *x*-axis represent 0.5 Mb. Ticks above the *x*-axis represent informative markers from WMD-MSG, i.e. markers flanking recombination events.
