# Supplementary material for: A Major Locus Controls a Genital Shape Difference Involved in Reproductive Isolation Between Drosophila yakuba and Drosophila santomea
Source: G3 (Bethesda). 2015 Oct 27;5(12):2893–901. doi: 10.1534/g3.115.023481 (PMC4683660; doi:10.1534/g3.115.023481)
Supplement: Supporting Information [file supp_g3.115.023481_FigureS8.pdf]

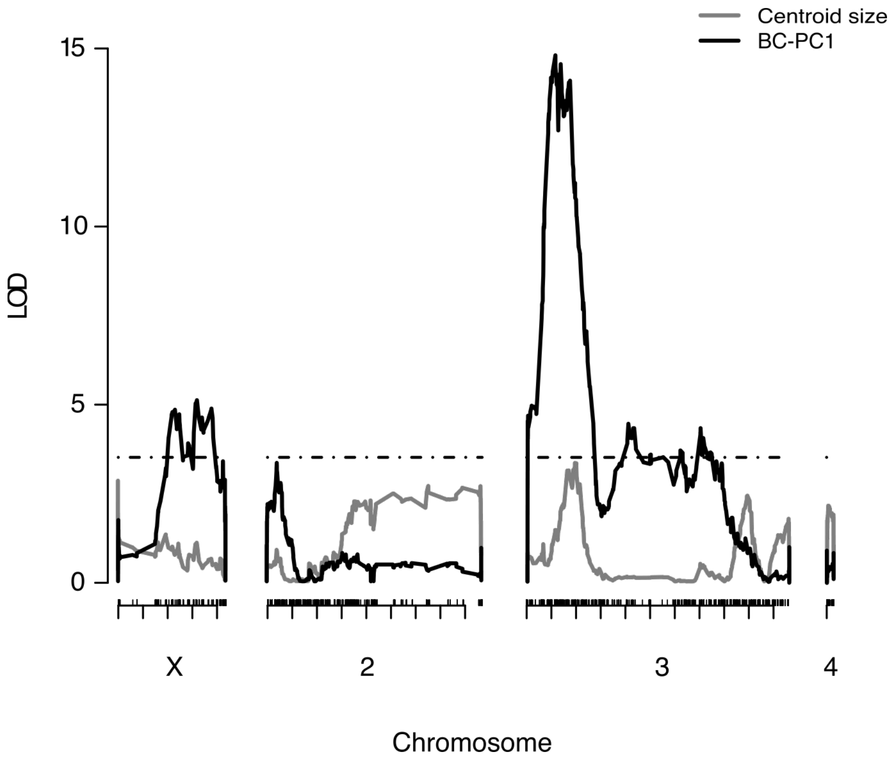

Figure S8. QTL analysis of BC-PC1 and centroid size in the *D. santomea* backcross. LOD profiles from a Haley-Knott regression analysis for BC-PC1 (generalized Procrustes analysis performed only on the backcross progeny) and for centroid size. The dotted line represents the 1% significance threshold (same for BC-PC1 and centroid size). Coordinates are given in mega-base pairs. Distance between two ticks below the *x*-axis represent 0.5 Mb. Ticks above the *x*-axis represent informative markers from WMD-MSG.
