# Supplementary material for: A Major Locus Controls a Genital Shape Difference Involved in Reproductive Isolation Between Drosophila yakuba and Drosophila santomea
Source: G3 (Bethesda). 2015 Oct 27;5(12):2893–901. doi: 10.1534/g3.115.023481 (PMC4683660; doi:10.1534/g3.115.023481)
Supplement: Supporting Information [file supp_g3.115.023481_FigureS9.pdf]

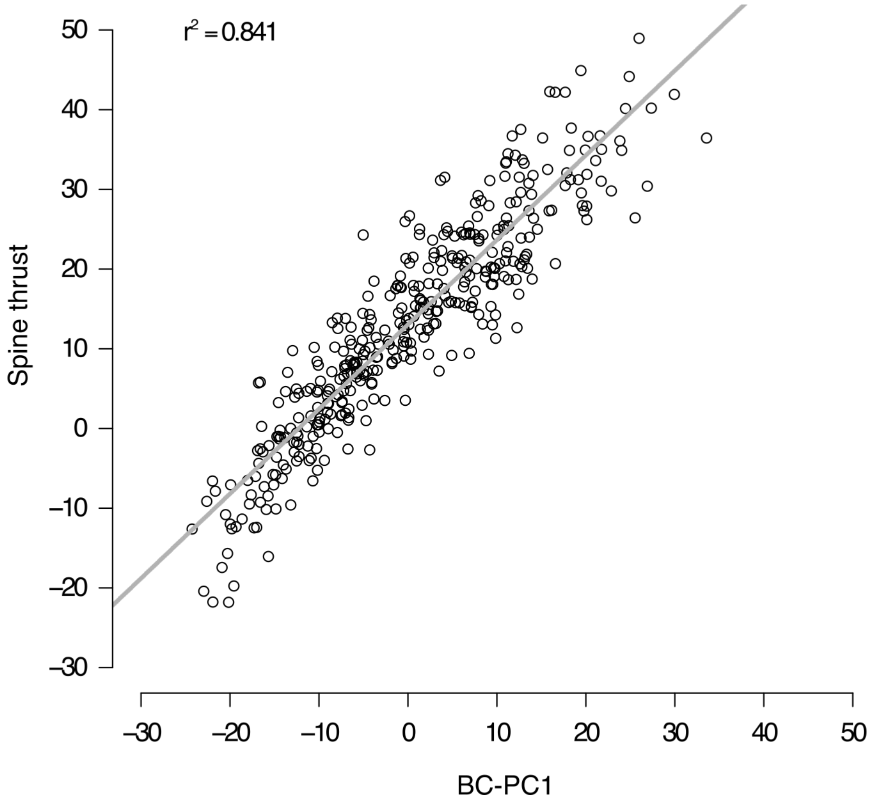

Figure S9. Correlation between BC-PC1 and "spine thrust". Each point represents one *D. santomea* backcross progeny individual.
