# Supplementary material for: A Major Locus Controls a Genital Shape Difference Involved in Reproductive Isolation Between Drosophila yakuba and Drosophila santomea
Source: G3 (Bethesda). 2015 Oct 27;5(12):2893–901. doi: 10.1534/g3.115.023481 (PMC4683660; doi:10.1534/g3.115.023481)
Supplement: Supporting Information [file supp_g3.115.023481_FileS7.pdf]

MSG by WMD
(Multiplexed Shotgun Genotyping by Whole Genome Amplification using Manta Polymerase with Degenerate Primers)

David Stern
1 Mar. 2014

What is it good for?

This protocol allows rapid preparation of reduced-representation libraries suitable for Multiplexed Shotgun Genotyping. These libraries do not seem to provide even coverage of the entire genome and, therefore are not suitable for whole-genome resequencing.

What is the basic principle?

A nicking endonuclease generates single-strand breaks in DNA which serves as a template for a mesophilic DNA polymerase with strand displacement activity. The ssDNA generated by the polymerase serves as a template for oligonucleotides with a 5' adaptor sequence and a 3' 12 bp degenerate sequence. At intermediate temperatures the degenerate ends of these oligos serve as primers for the mesophilic polymerase. Eventually, these adaptor labeled strands become displaced and primed anew by another adaptor-degenerate primer, generating a strand with adaptor sequences at both ends. In a second step, these products serve as templates for PCR using primers with a 5' sequence compatible with the required sequencing platform and a 3' end identical to the adaptor sequences.

What are the key variables?

1 - The oligonucleotides used in the isothermal amplification must carry a 5' C18 modification. I have tried many other modifications of oligos, and this is the only one that reliably prevents amplification of oligos in the absence of template DNA.

2 - The DNA polymerase must be strand displacing and have significant activity at 50°C. I use Manta DNA polymerase from Enzymatics. This is a version of BST DNA polymerase, Large Fragment. Unlike the enzyme from other manufacturers, this product is not contaminated with DNA and Enzymatics provides extremely generous aliquots for your money.

3 - You must use Nt.Bpu10I nicking enzyme from Fermentas (ThermoFisher). I have tried almost all other nicking enzymes on the market. Many are contaminated with DNA and of the remainder, this is the only one with appropriate activity at 50°C.

4 - You must use Buffer R, supplied with the Nt.Bpu10I, for the isothermal amplification. The Manta polymerase works fine in this buffer. The nicking enzyme will not work in the

Manta buffer. I have tried to modify Buffer R to make it more like the Manta polymerase and these all performed worse than straight Buffer R.

What are the advantages of the method?

1 - Very little hands-on time
2 - Rapid library preparation, usually < 1 day from flies to library
3 - Ability to use poor-quality, low concentration DNA
4 - The two step protocol allows adding two sets of barcodes, for extremely high multiplexing with a small number of primers.
5 - This protocol may provide more even barcode representation than classic MSG.

What are the deficiencies of the method?

1 - This method does not provide even genomic coverage.
2 - In practice, usually multiple cycles of PCR are required (20-35), leading to clonal amplification, which should be taken into account in downstream applications.
3 - Depending on the sequencing method, from ~12 to ~35 bp of adaptor sequences must be trimmed from the reads.
4 - In practice, the method works in my hands *only* with the specified products from the specified manufacturers. I have tried many other combinations. The most common problem is that most manufacturers do not produce enzymes of sufficient purity. Usually, enzymes, especially those from NEB, are contaminated with low levels of DNA. I have found that other enyzme/oligo combinations also fail. When I follow the protocol below, I have had 100% success.

This protocol works, of course, with high quality DNA, but it also works with low quality DNA. The protocol appears to be sensitive to low levels of EtOH. Ensure EtOH is removed completely from your DNA preps. Below, I provide the full protocol from fly to library in a single day. If you have trouble, you may want to make higher quality DNA.

1 - Make DNA

I use the Zymo Quick-gDNA MicroPrep for a small number of flies and the Zymo ZR-96 Quick-gDNA for plates of 96 flies at a time.

For small number of flies:

In a 2mL round bottom Eppendorf tube, add 1 metal BB, 1 fly, and 100-200 uL of Zymo gDNA grinding buffer. Grind in Disruptor Genie for 10 minutes.

Transfer liquid (with miscellaneous fly bits and all) to a Zymo silicon spin column and follow protocol.

For 96-well plate of flies:

Prepare round-bottom 96-well plate with 1 BB per well. Add 100uL of Zymo grinding buffer and transfer individual flies to each well. Seal plate and run in seed grinder for 7 minutes.

I have found you can also prepare samples for WMD without purifying the gDNA.

Collect an individual fly into a 2 mL Eppendorf tube with 1 BB and place at -80°C for 5 minutes.
Add 100 uL SB + ProtK
Add 1 BB and grind on Disruptor Genie.

Spin at top speed 1 minute

Dispense 50 uL of supernatant to small tubes. (May be optional, if can incubate in grinding plates.)

Incubate at 55°C 1 hour

95°C 2 minutes

1uL is sufficient for WMD.

2 - WGA with Degenerate Primers

In PCR tubes or PCR plate, add following.

	**Per Sample (uL)**	
DNA (~1-10ng)	5	1
5uM C18_RevAdaptor_BC	0.5	0.25
4 mM dNTP	0.5	0.25
Buffer R	1	0.5
Manta Pol. (40 U/uL)	0.25	0.125
Nt.Bpu10I (5 U/uL)	0.05	0.025
H_20	2.7	2.85
Total Volume	10	5

Place in thermocycler with following cycle.

The lower temperature allows the restriction enzyme to start cutting. The RE is active at 50° and the adaptors anneal and the Manta polymerizes DNA at 50°.

37°C 15 minutes
50°C 2 hours
80°C 20 minutes
10°C Forever

3 - Pool rows of barcoded reactions

Pool samples along rows into EDTA to stop reaction.
For large volume reactions, I sample 7uL per reaction into 5 uL of 0.5M EDTA.
For small volume reactions, I sample 2uL per reaction into 2 uL of 0.5M EDTA.

4 - AMPure clean samples

For large volume, clean 30uL of pooled reaction with 54 uL AMPure.
For small volume reactions, add 47 uL AMPure directly to the pooled sample (26uL).

****
At this point, the protocols differ slightly for libraries for Ion Torrent vs Illumina sequencing.

Ion Torrent library

4 I.T. - PCR with barcoded primers

I *strongly* recommend using Omni Klentaq from Enzymatics. This thermostable polymerase works considerably better than any other polymerase I have tried and consistently generates excellent amplification from the WMD shmears.

	**Per Reaction (uL)**	
Template (Pooled WMD)	5	2.5
5 uM A_BC_adaptor	0.5	0.25
5 uM P1_adaptor	0.5	0.25
4 mM dNTP	1	0.5
OmniKlenTaq 5X	4	2
OmniKlenTaq	0.5	0.25

	Per Reaction (uL)	
H_2O	8.5	4.25
Total Volume	20	10

Thermocycler program

98°C 30 sec
25X
    98°C 5 sec
    61°C 5 sec
    72°C 5 sec
4°C Hold

5 I.T. - Pool library, purify DNA & E-Gel purify library

Pool all barcoded samples.

AMPure clean the sample

Follow E-Gel protocol to size select for your sequencing platform. For the IT, I select the sample after the 350bp band of the ladder has fully entered the sample well.

6 I.T. - Optional, but highly recommended, quantification and quality control check on Bioanalyzer

If it worked, you should see a peak at ~ 250-300bp. These libraries behave differently than classic library protocols and I have found consistently that these libraries have a higher proportion of fragments with correct sequencing termini than shear-and-ligate protocols. I therefore usually multiple the dilution by ~3 to get a good dilution of products for IT sequencing. For example, if my library is 600 pMol, I use a dilution factor of 45.

7 I.T. - Optional, if low yield. Ethanol precipitation to concentrate sample

Add 3M NaAcetate pH 5.2 to final concetration of 10%. Add 2.5X volume 100% ethanol. Place at -20°C overnight. Spin at high speed for 30 minutes. Wash with 1mL 70% ethanol. Spin high speed 30 minutes. Resuspend DNA in desired volume (I use 10% of original volume). Quantify, etc.

****

Illumina library

4 III - PCR with barcoded FC2 RevAdapt and FC1 RevAdapt primers

I *strongly* recommend using Omni Klentaq from Enzymatics. This thermostable polymerase works considerably better than any other polymerase I have tried and consistently generates excellent amplification from the WMD shmears. Protocol was developed with 25 cycles of PCR, but fewer cycles would probably work.

	**Per Rxn (uL)**	
Template (Pooled WMD)	5	2.5
5 uM FC2_BC_RevAdapt	0.5	0.25
5 uM FC1_RevAdapt	0.5	0.25
4 mM dNTP	1	0.5
OmniKlenTaq 5X	4	2
OmniKlenTaq	0.5	0.25
H_20	8.5	4.25
Total Volume	20	10

Thermocycler program

98°C 30 sec
20 X
 98°C 5 sec
 61°C 5 sec
 72°C 5 sec
4°C Hold

5 III - Pool library, purify DNA

Pool all barcoded samples.
AMPure purify 40 uL of sample (72uL AMPure).

(Other methods of purification can also be used, such as Zymo Clean and Concentrator.)

6 III - PCR with FC2 and FC1 primers

	Per Reaction
Template (Pooled PCR1)	20
5 uM FC2	1
5 uM FC1	1
4 mM dNTP	2
OmniKlenTaq 5X Buffer	8
OmniKlenTaq	1
H_20	7
Total Volume	40

Thermocycler program

98°C 30 sec
10X
98°C 5 sec
65°C 10 sec (**Note this is a higher temp than first PCR!**)
72°C 30 sec
4°C Hold

I often run 10uL of the reaction to ensure amplification has worked and produced a shmear of the correct distribution.

7 III - Purify library

Purify the amplified sample away from primers. There are several methods available
1- AMPure clean 30uL with 54uL of AMPure
2 - Clean up with Zymo Zlean & Concentrator 5 kit

If the gel of PCR2 revealed a primer-dimer band, I often run a new gel (or a BioAnalyzer chip) with the purified sample to confirm removal of the primer dimers.

Submit this sample for sequencing.

Sequences of required primers

For Ion Torrent sequencing

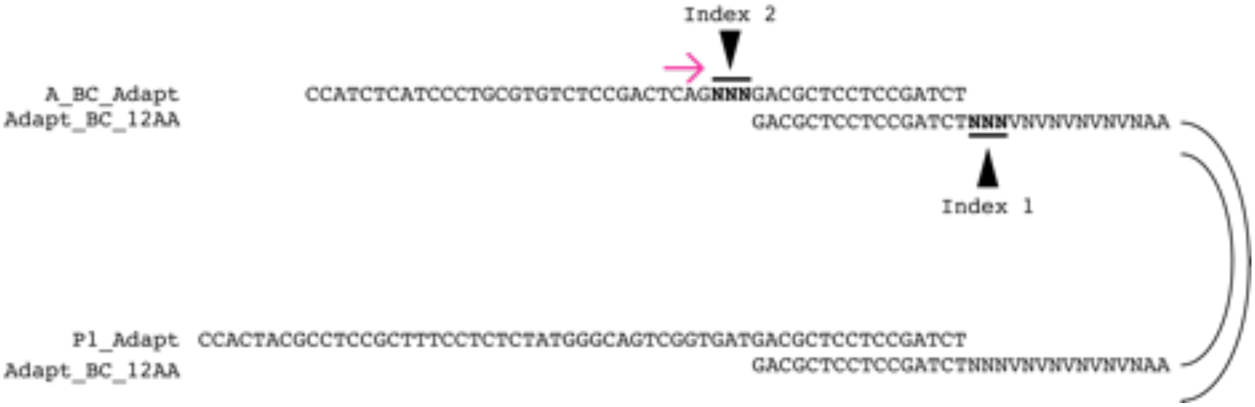

IT WMD oligos	Barcode	Primer
C18_Adapt_3BC_12AA_01	AAT	T/ISP18/GACGCTCCTCCGATCTAATVNVNVNVNVNAA
C18_Adapt_3BC_12AA_02	ACT	T/ISP18/GACGCTCCTCCGATCTACTVNVNVNVNVNAA
C18_Adapt_3BC_12AA_03	ATT	T/ISP18/GACGCTCCTCCGATCTATTVNVNVNVNVNAA
C18_Adapt_3BC_12AA_06	GCT	T/ISP18/GACGCTCCTCCGATCTGCTVNVNVNVNVNAA
C18_Adapt_3BC_12AA_07	GTT	T/ISP18/GACGCTCCTCCGATCTGTTVNVNVNVNVNAA
C18_Adapt_3BC_12AA_08	GGT	T/ISP18/GACGCTCCTCCGATCTGGTVNVNVNVNVNAA
C18_Adapt_3BC_12AA_13	ACA	T/ISP18/GACGCTCCTCCGATCTACAVNVNVNVNVNAA
C18_Adapt_3BC_12AA_14	ATA	T/ISP18/GACGCTCCTCCGATCTATAVNVNVNVNVNAA
C18_Adapt_3BC_12AA_15	AGA	T/ISP18/GACGCTCCTCCGATCTAGAVNVNVNVNVNAA
C18_Adapt_3BC_12AA_16	AGT	T/ISP18/GACGCTCCTCCGATCTAGTVNVNVNVNVNAA
C18_Adapt_3BC_12AA_17	CAC	T/ISP18/GACGCTCCTCCGATCTCACVNVNVNVNVNAA
C18_Adapt_3BC_12AA_18	CTA	T/ISP18/GACGCTCCTCCGATCTCTAVNVNVNVNVNAA

IT PCR BC primers	Barcode	Sequence
A_BC_Adapt_1	ACA	CCATCTCATCCCTGCGTGTCTCCGACTCAGACAGACGCTCCTCCGATCT
A_BC_Adapt_2	ACT	CCATCTCATCCCTGCGTGTCTCCGACTCAGACTGACGCTCCTCCGATCT
A_BC_Adapt_3	AAC	CCATCTCATCCCTGCGTGTCTCCGACTCAGAACGACGCTCCTCCGATCT

IT PCR BC primers	Barcode	Sequence
A_BC_Adapt_4	TCA	CCATCTCATCCCTGCGTGTCTCCGACTCAGTCAGACGCTCCTCCGATCT
A_BC_Adapt_5	TAT	CCATCTCATCCCTGCGTGTCTCCGACTCAGTATGACGCTCCTCCGATCT
A_BC_Adapt_6	TGC	CCATCTCATCCCTGCGTGTCTCCGACTCAGTGCGACGCTCCTCCGATCT
A_BC_Adapt_7	CTA	CCATCTCATCCCTGCGTGTCTCCGACTCAGCTAGACGCTCCTCCGATCT
A_BC_Adapt_8	CTC	CCATCTCATCCCTGCGTGTCTCCGACTCAGCTCGACGCTCCTCCGATCT

For Illumina sequencing

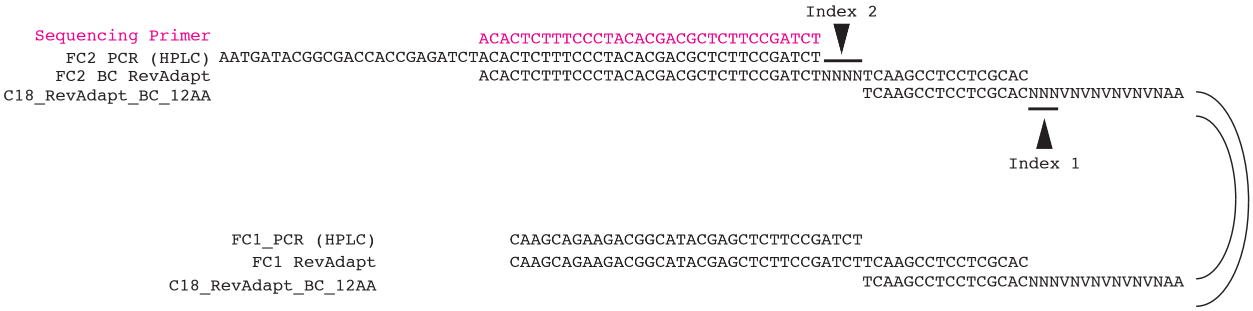

12 Adapt_BC_12AA
32 Ill_Adapt_1_BC
= 384 barcoded individuals

Illumina WMD oligos	Barcode	Primer
C18 rev-adapt 01	tca	T/ISP18/CAAGCCTCCTCGCACtcaVNVNVNVNVNAA
C18 rev-adapt 02	tga	T/ISP18/CAAGCCTCCTCGCACtgaVNVNVNVNVNAA
C18 rev-adapt 03	acc	T/ISP18/CAAGCCTCCTCGCACaccVNVNVNVNVNAA
C18 rev-adapt 04	cca	T/ISP18/CAAGCCTCCTCGCACccaVNVNVNVNVNAA
C18 rev-adapt 05	cta	T/ISP18/CAAGCCTCCTCGCACctaVNVNVNVNVNAA
C18 rev-adapt 06	tgt	T/ISP18/CAAGCCTCCTCGCACtgtVNVNVNVNVNAA
C18 rev-adapt 07	cgc	T/ISP18/CAAGCCTCCTCGCACcgcVNVNVNVNVNAA
C18 rev-adapt 08	ata	T/ISP18/CAAGCCTCCTCGCACataVNVNVNVNVNAA
C18 rev-adapt 09	ctg	T/ISP18/CAAGCCTCCTCGCACctgVNVNVNVNVNAA
C18 rev-adapt 10	ccg	T/ISP18/CAAGCCTCCTCGCACccgVNVNVNVNVNAA

Illumina WMD oligos	Barcode	Primer
C18 rev-adapt 11	cag	T/ISP18/CAAGCCTCCTCGCACcagVNVNVNVNVNAA
C18 rev-adapt 12	tgg	T/ISP18/CAAGCCTCCTCGCACtggVNVNVNVNVNAA

Illumina BC primers	Barcode	Sequence
IllFC2_Adapt_1	gacc	ACACTCTTTCCCTACACGACGCTCTTCCGATCTgaccTCAAGCCTCCTCGCAC
IllFC2_Adapt_2	cgccc	ACACTCTTTCCCTACACGACGCTCTTCCGATCTcgcccTCAAGCCTCCTCGCAC
IllFC2_Adapt_3	actgag	ACACTCTTTCCCTACACGACGCTCTTCCGATCTactgagTCAAGCCTCCTCGCAC
IllFC2_Adapt_4	gtct	ACACTCTTTCCCTACACGACGCTCTTCCGATCTgtctTCAAGCCTCCTCGCAC
IllFC2_Adapt_5	tatcc	ACACTCTTTCCCTACACGACGCTCTTCCGATCTtatccTCAAGCCTCCTCGCAC
IllFC2_Adapt_6	gctacg	ACACTCTTTCCCTACACGACGCTCTTCCGATCTgctacgTCAAGCCTCCTCGCAC
IllFC2_Adapt_7	cacg	ACACTCTTTCCCTACACGACGCTCTTCCGATCTcacgTCAAGCCTCCTCGCAC
IllFC2_Adapt_8	caaag	ACACTCTTTCCCTACACGACGCTCTTCCGATCTcaaagTCAAGCCTCCTCGCAC
IllFC2_Adapt_9	gtgcct	ACACTCTTTCCCTACACGACGCTCTTCCGATCTgtgcctTCAAGCCTCCTCGCAC
IllFC2_Adapt_10	gatt	ACACTCTTTCCCTACACGACGCTCTTCCGATCTgattTCAAGCCTCCTCGCAC
IllFC2_Adapt_11	ttgta	ACACTCTTTCCCTACACGACGCTCTTCCGATCTttgtaTCAAGCCTCCTCGCAC
IllFC2_Adapt_12	cgcaca	ACACTCTTTCCCTACACGACGCTCTTCCGATCTcgcacaTCAAGCCTCCTCGCAC
IllFC2_Adapt_13	aacg	ACACTCTTTCCCTACACGACGCTCTTCCGATCTaacgTCAAGCCTCCTCGCAC
IllFC2_Adapt_14	gtcta	ACACTCTTTCCCTACACGACGCTCTTCCGATCTgtctaTCAAGCCTCCTCGCAC

Illumina BC primers	Barcode	Sequence
IllFC2_Adapt_15	aaaaat	ACACTCTTTCCCTACACGACGCTCTTCCGATCTaaaaatTCAAGCCTCCTCGCAC
IllFC2_Adapt_16	ctga	ACACTCTTTCCCTACACGACGCTCTTCCGATCTctgaTCAAGCCTCCTCGCAC
IllFC2_Adapt_17	gacgt	ACACTCTTTCCCTACACGACGCTCTTCCGATCTgacgtTCAAGCCTCCTCGCAC
IllFC2_Adapt_18	cgcaac	ACACTCTTTCCCTACACGACGCTCTTCCGATCTcgcaacTCAAGCCTCCTCGCAC
IllFC2_Adapt_19	ggtc	ACACTCTTTCCCTACACGACGCTCTTCCGATCTggtcTCAAGCCTCCTCGCAC
IllFC2_Adapt_20	tgtaa	ACACTCTTTCCCTACACGACGCTCTTCCGATCTtgtaaTCAAGCCTCCTCGCAC
IllFC2_Adapt_21	ttcccg	ACACTCTTTCCCTACACGACGCTCTTCCGATCTttcccgTCAAGCCTCCTCGCAC
IllFC2_Adapt_22	gaat	ACACTCTTTCCCTACACGACGCTCTTCCGATCTgaatTCAAGCCTCCTCGCAC
IllFC2_Adapt_23	aggtc	ACACTCTTTCCCTACACGACGCTCTTCCGATCTaggtcTCAAGCCTCCTCGCAC
IllFC2_Adapt_24	agcaca	ACACTCTTTCCCTACACGACGCTCTTCCGATCTagcacaTCAAGCCTCCTCGCAC
IllFC2_Adapt_25	gact	ACACTCTTTCCCTACACGACGCTCTTCCGATCTgactTCAAGCCTCCTCGCAC
IllFC2_Adapt_26	gttat	ACACTCTTTCCCTACACGACGCTCTTCCGATCTgttatTCAAGCCTCCTCGCAC
IllFC2_Adapt_27	atccac	ACACTCTTTCCCTACACGACGCTCTTCCGATCTatccacTCAAGCCTCCTCGCAC
IllFC2_Adapt_28	tgcc	ACACTCTTTCCCTACACGACGCTCTTCCGATCTtgccTCAAGCCTCCTCGCAC
IllFC2_Adapt_29	cccgt	ACACTCTTTCCCTACACGACGCTCTTCCGATCTcccgtTCAAGCCTCCTCGCAC
IllFC2_Adapt_30	aatcct	ACACTCTTTCCCTACACGACGCTCTTCCGATCTaatcctTCAAGCCTCCTCGCAC
IllFC2_Adapt_31	ttga	ACACTCTTTCCCTACACGACGCTCTTCCGATCTttgaTCAAGCCTCCTCGCAC

Illumina BC primers	Barcode	Sequence
IllFC2_Adapt_32	attta	ACACTCTTTCCCTACACGACGCTCTTCCGATCTatttaTCAAGCCTCCTCGCAC
