# Supplementary material for: A Major Locus Controls a Genital Shape Difference Involved in Reproductive Isolation Between Drosophila yakuba and Drosophila santomea
Source: G3 (Bethesda). 2015 Oct 27;5(12):2893–901. doi: 10.1534/g3.115.023481 (PMC4683660; doi:10.1534/g3.115.023481)
Supplement: Supporting Information [file supp_g3.115.023481_FigureS1.pdf]

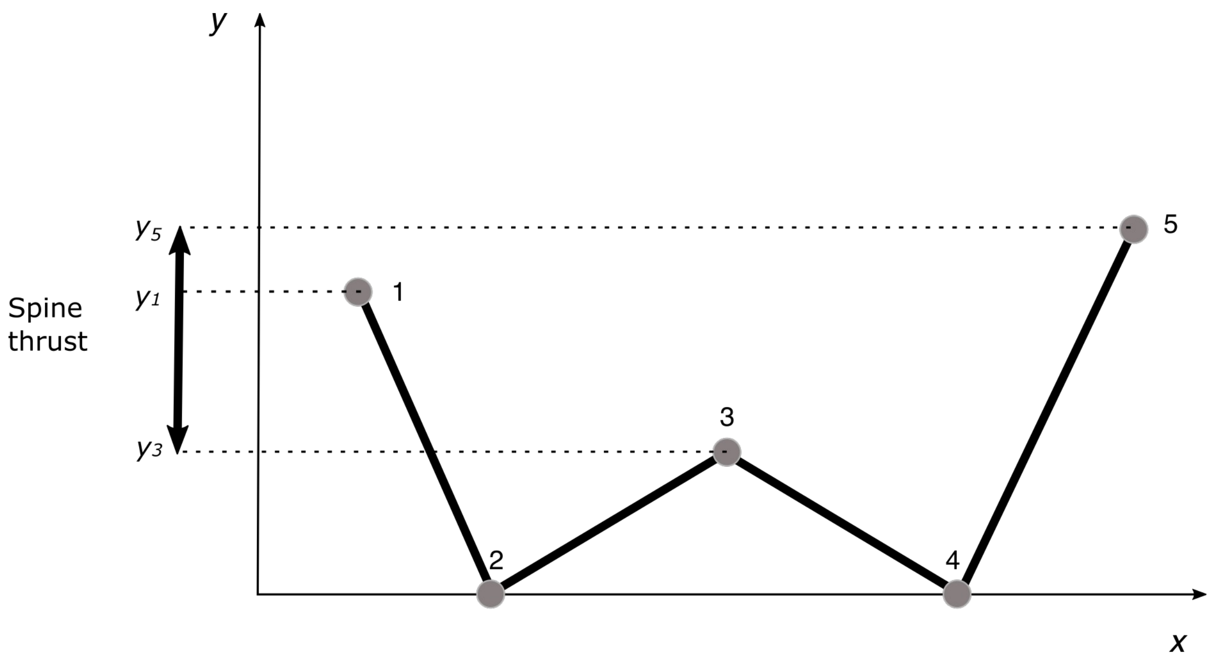

Figure S1. Illustration of the "spine thrust" measure for a *D. santomea* backcross individual. For each individual "spine thrust" is computed as max(*y1*;*y*5)-*y*3 where *y*i is the coordinate of landmark i when the *x*-axis is defined as the axis passing by landmarks 2 and 4, oriented from 2 to 4, and with the *y*-axis defined so that (*x*,*y*) is an oriented orthonormal basis.
