# Supplementary material for: A Major Locus Controls a Genital Shape Difference Involved in Reproductive Isolation Between Drosophila yakuba and Drosophila santomea
Source: G3 (Bethesda). 2015 Oct 27;5(12):2893–901. doi: 10.1534/g3.115.023481 (PMC4683660; doi:10.1534/g3.115.023481)
Supplement: Supporting Information [file supp_g3.115.023481_FigureS2.pdf]

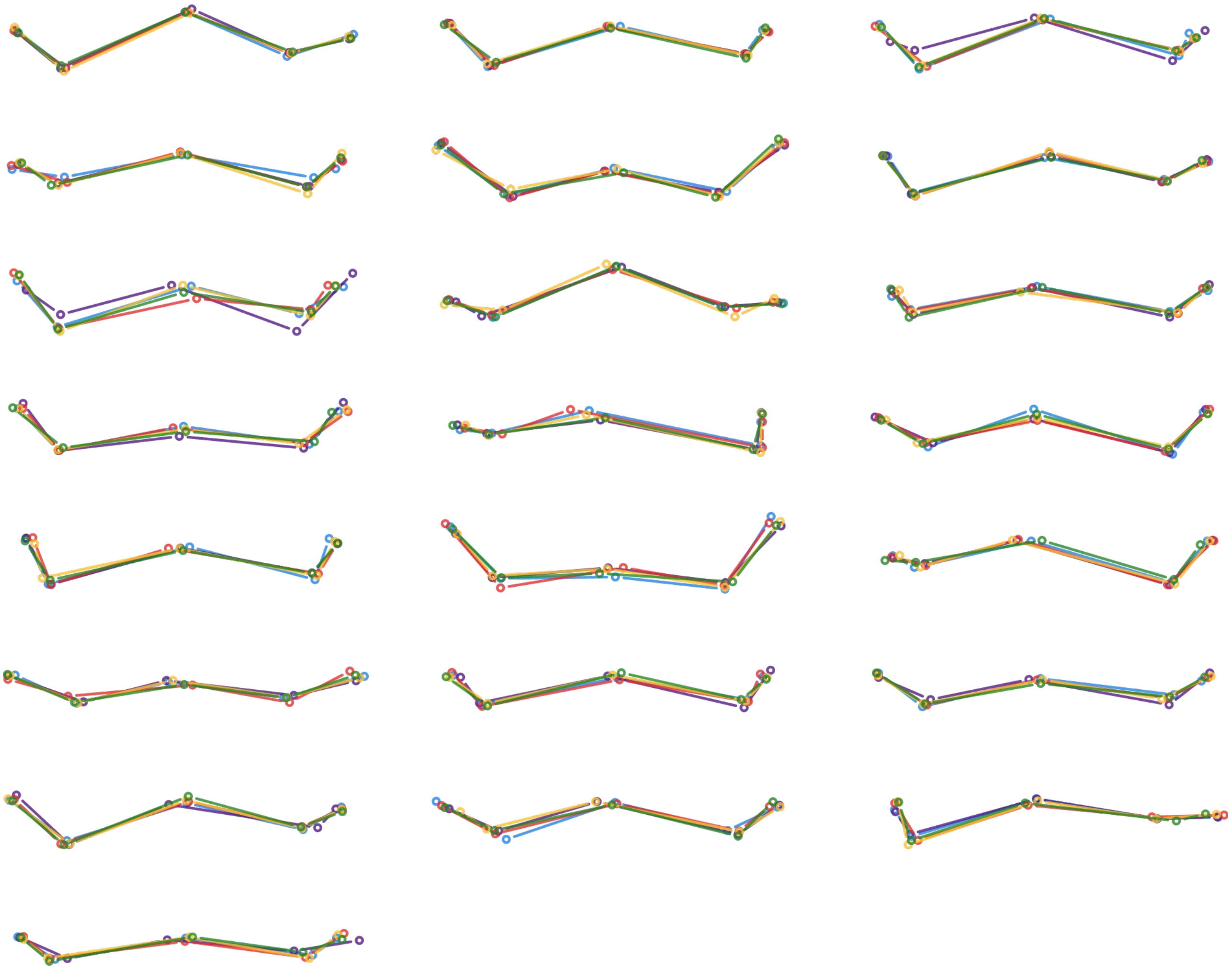

Figure S2. Repeatability of configuration acquisition for *D. santomea* individuals. 22 individuals are shown. For each individual, five sessions of sample mounting, picture acquisition and landmark acquisition were done. Each color represents one session. Configurations where aligned with a generalized Procrustes superimposition for each individual.
