# Supplementary material for: A Major Locus Controls a Genital Shape Difference Involved in Reproductive Isolation Between Drosophila yakuba and Drosophila santomea
Source: G3 (Bethesda). 2015 Oct 27;5(12):2893–901. doi: 10.1534/g3.115.023481 (PMC4683660; doi:10.1534/g3.115.023481)
Supplement: Supporting Information [file supp_g3.115.023481_FigureS3.pdf]

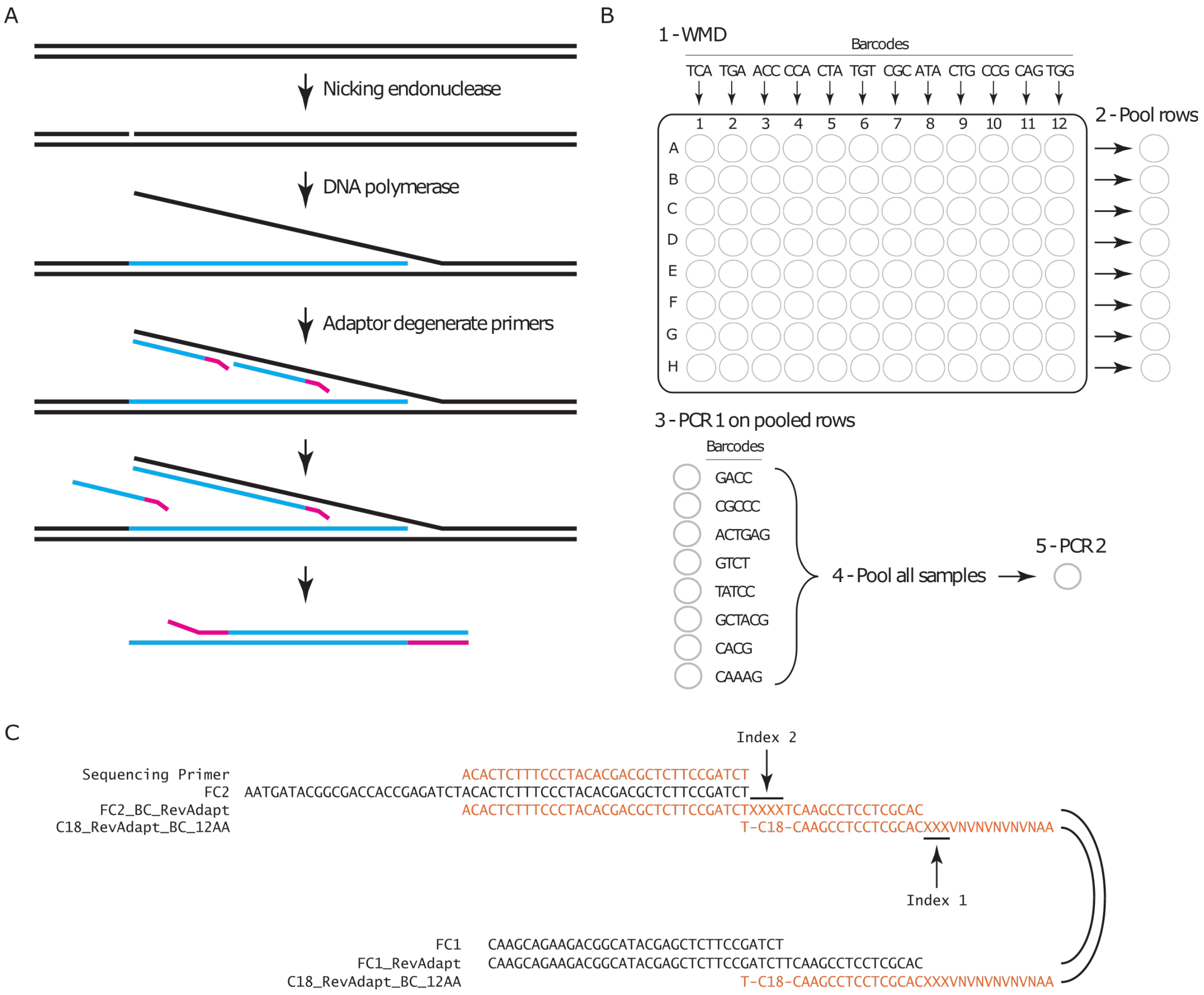

Figure S3. Whole genome amplification using manta polymerase with degenerate primers (WMD). (a) A schematic of the critical steps during WMD. From top to bottom, first, DNA is cleaved site-specifically on a single strand using the engineered restriction enzyme, Nt.Bpu10I. Second, the mesothermic DNA polymerase Manta 1.0 DNA polymerase initiates DNA synthesis from the 3’ end of the nicked DNA (blue strand). Manta polymerase has strong strand displacement activity, which results in the release of a single-strand of DNA. Third, adaptor oligonucleotides containing a partially degenerate 3’ end (pink) hybridize to multiple regions on the single-stranded DNA and provide templates for DNA synthesis (blue) by Manta DNA polymerase. Fourth, the strand displacement activity of Manta polymerase leads to release of single-stranded fragments containing an adaptor oligonucleotide at their 5’ end. Finally, these single-stranded fragments serve as templates for priming by additional adaptor oligonucleotides. Synthesis of the complementary strand produces single stranded products (the top strand of the final product) that are suitable for addition of platform-specific adaptor sequences by polymerase chain reaction. In practice, the three reagents (restriction enzyme, mesothermic DNA polymerase, and modified adaptor oligonucleotides) are combined with DNA and incubated in a single tube, first at 37°C to ensure restriction digestion and then at 50°C to facilitate DNA polymerization. The restriction

enzyme used here, Nt.Npu10I, may continue to cut at 50°C and is inactivated by an 80°C step at the end of the protocol. (b) Workflow for a single plate of 96 samples. First, WMD is performed on samples with a separate barcoded C18_RevAdapt_BC_12AA oligonucleotide used in each column of a 96-well plate. Second, samples are pooled by row. Third, PCR is performed on samples of pooled rows to introduce sequencing platform-specific sequences and a second barcode of variable length. The variable length barcode increases the fidelity of Illumina-based sequencing. Finally, all samples are pooled, cleaned to eliminate residual primers, and a final PCR is performed on the entire sample with platform-specific primers. The sample is now ready for purification, quantification, and sequencing. (c) Diagram illustrating complementarity of the adaptor oligonucleotides containing partially degenerate sequences at their 3' end (pink), and the oligonucleotides used for sequencing on an Illumina platform. The first round of PCR is performed using the FC2_BC_RevAdapt and FC1_RevAdapt oligonucleotides to add a second index sequence to the sequenced end of the fragments. A second round of PCR is performed with FC2 and FC1 oligonucleotides to produce the final library.
