# Supplementary material for: A Major Locus Controls a Genital Shape Difference Involved in Reproductive Isolation Between Drosophila yakuba and Drosophila santomea
Source: G3 (Bethesda). 2015 Oct 27;5(12):2893–901. doi: 10.1534/g3.115.023481 (PMC4683660; doi:10.1534/g3.115.023481)
Supplement: Supporting Information [file supp_g3.115.023481_FigureS5.pdf]

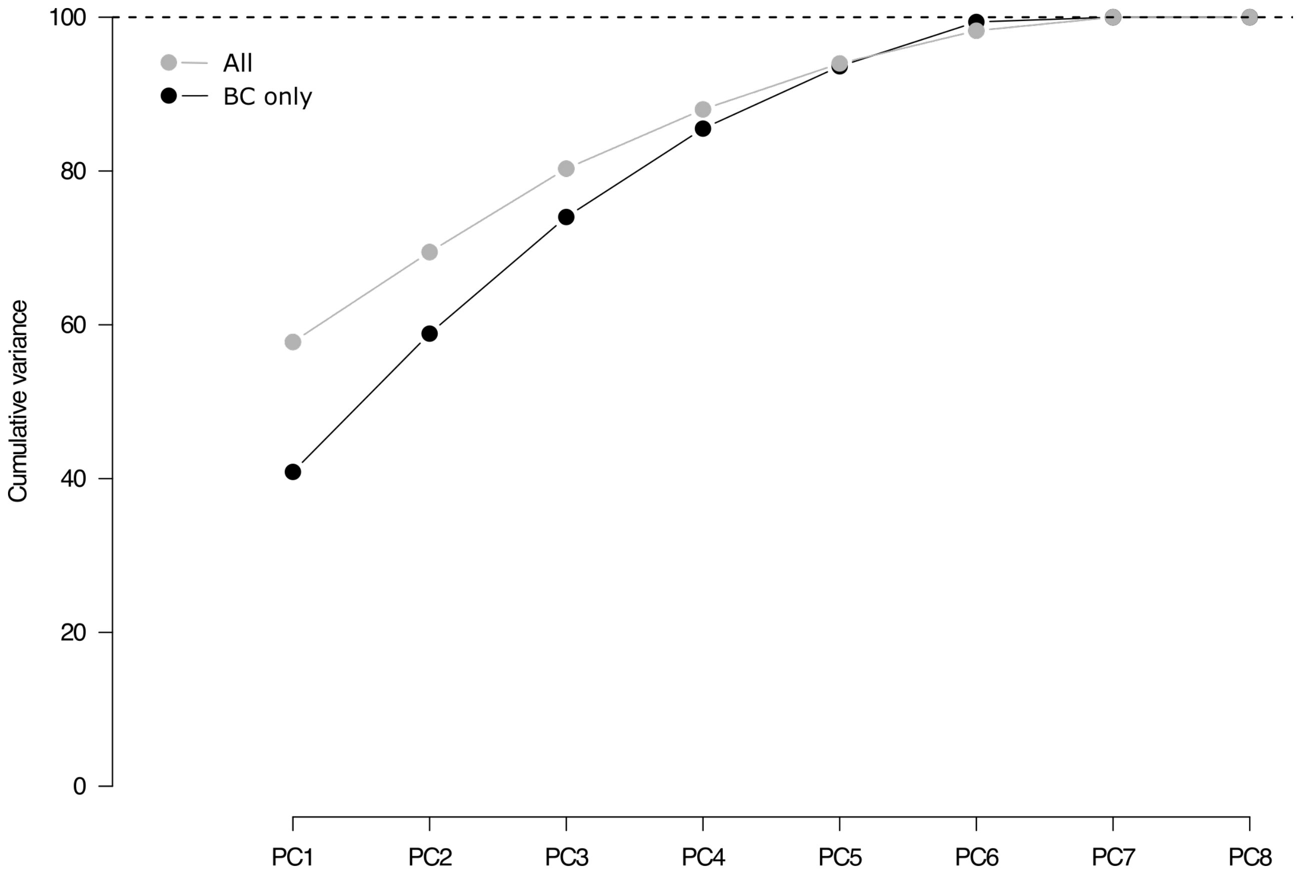

Figure S5. Cumulative variance explained by principal component analysis of the generalized Procrustes analysis of ventral branch landmark configurations. Principal component analysis (PCA) performed only on the backcross progeny (gray) and on the full dataset comprising the two parental species, F1 hybrids and backcross progeny (black) are shown. For the full dataset PCA, the first eight principal components explain 57.8, 11.7, 10.8, 7.7, 6.0, 4.3, 1.8, and $9.1\text{x}10^{-10}$ percent of the variance. For the Backcross PCA, the first eight principal components explain 40.9, 18.0, 15.2, 11.5, 8.0, 5.8, 0.6, and $3.5\text{x}10^{-8}$ percent of the variance.
